# Senataxin prevents replicative stress induced by the Myc oncogene

**DOI:** 10.1038/s41419-025-07485-4

**Published:** 2025-03-19

**Authors:** Silvia Sberna, Marco Filipuzzi, Nicola Bianchi, Ottavio Croci, Federica Fardella, Chiara Soriani, Sara Rohban, Sara Carnevali, Alessandra Alberta Albertini, Nicola Crosetto, Simona Rodighiero, Arianna Chiesa, Laura Curti, Stefano Campaner

**Affiliations:** 1 Center for Genomic Science of IIT, CGS@SEMM (Istituto Italiano di Tecnologia at European School of Molecular Medicine), Fondazione Istituto Italiano di Tecnologia (IIT), 20139 Milan, Italy; 2 Imaging Unit, Department of Experimental Oncology, IEO, European Institute of Oncology IRCCS, Milan, Italy; 3 Human Technopole, Viale Rita Levi-Montalcini 1, 20157 Milan, Italy; 4 Department of Microbiology, Tumor and Cell Biology, Karolinska Institutet, Stockholm, SE 17165 Sweden; 5 Science for Life Laboratory, Tomtebodavägen 23A, Solna, SE 17165 Sweden; 6 Department of Molecular Medicine, University of Padua, Padua, Italy

**Keywords:** Checkpoints, Oncogenes

## Abstract

Replicative stress (RS) is emerging as a promising therapeutic target in oncology, yet full exploitation of its potential requires a detailed understanding of the mechanisms and genes involved. Here, we investigated the RNA helicase Senataxin (SETX), an enzyme that resolves RNA-DNA hybrids and R-loops, to address its role in preventing RS by oncogenic Myc. Upon Myc activation, silencing of SETX led to selective engagement of the DNA damage response (DDR) and robust cytotoxicity. Pharmacological dissection of the upstream kinases regulating the DDR uncovered a protective role of the ATR pathway, that once inhibited, boosted SETX driven-DDR. While SETX loss did not lead to a genome-wide increase of R-loops, mechanistic analyses revealed enhanced R-loops localized at DDR-foci and newly replicated genomic loci, compatible with a selective role of SETX in resolving RNA-DNA hybrids to alleviate Myc-induced RS. Genome-wide mapping of DNA double-strand breaks confirmed that SETX silencing exacerbated DNA damage at transcription-replication conflict (TRC) regions at early replicated sites. We propose that SETX prevents Myc-induced TRCs by resolving transcription-associated R-loops that encounter the replisome. The identification of SETX as a genetic liability of oncogenic Myc opens up new therapeutic options against aggressive Myc-driven tumors.

## Introduction

c-Myc (henceforth Myc) is a transcription factor of the leucine-zipper basic helix-loop-helix family that heterodimerizes with Max to activate or repress genes in a context-dependent manner [1–3]. In physiological conditions, Myc expression is activated by growth factor signaling and leads to the transactivation of target genes controlling cell cycle progression and regulation of cell metabolism. Once activated in cancer cells, Myc extends its regulation to thousands of genes, thus gaining control of basic processes required for self-sustainment and proliferation of cancer cells and repressing cell differentiation [4]. The hijacking of a broad range of cellular genes linked to diverse cellular responses also leads to the activation of intrinsic tumor suppressive pathways, such as Myc-induced apoptosis and replicative stress (RS) [5–7]. Indeed, as observed with other oncogenes, deregulation of Myc alters various mechanisms of cell cycle control, in particular during S-phase, thus leading to RS associated with genomic instability [8, 9]. Intrinsic RS can be boosted to drive strong cytotoxic responses in Myc-driven cancer cells, opening up an avenue for targeted intervention based on the exacerbation of the DNA damage response (DDR), as shown by targeting the ATR/CHK1 pathway, RAD21, BRCA1, TOP1 [10–13], and more recently CDK12 [14].

Recent data suggests that overexpression of MYC or its paralogues may put cells on the verge of rampant genomic instability due to enhanced transcription-replication conflicts (TRCs). This is in part due to the ability of Myc to anticipate the initiation of DNA synthesis, thus creating a clash between the replication of late G1 transcribed genes and their transcription [8].

R-loops are among the emerging factors causally linked to oncogene-induced RS and genomic instability [15–19]. R-loops are structures generated by stable RNA-DNA hybrids that form behind RNA polymerase II as transcription progresses. In the presence of R-loops, the displacement of the non-template DNA strand may impose a physical barrier that hampers DNA replication, leading to the collapse of the replication fork and the generation of DNA double-strand breaks (DSBs). Alternatively, DSBs can also originate from the processing of R-loops by nucleases of the Nucleotide Excision Repair pathway (NER) [20]. R-loops can also be resolved by pathways that do not involve the introduction of DNA breaks, such as the unwinding of RNA-DNA hybrids by helicases or the enzymatic digestion of the RNA component by RNAseH [16, 21], indicating that the modality and the efficiency of R-loops resolution are critical to the maintenance of genomic integrity.

Senataxin (SETX) is one of the best-characterized RNA-DNA helicases known to resolve R-loops [22, 23]. SETX is mutated in neurological disorders, such as oculomotor apraxia type 2 [24–27], and in autosomal dominant amyotrophic lateral sclerosis type 4 [24, 28]. It is reported to facilitate gene transcription by resolving R-loops at promoter pausing sites [23, 29] and by promoting transcription termination at poly(A) sites, where it favors the recruitment of the 5’-3’ exonuclease Xrn2, which degrades the 3’ end of the nascent transcripts [29–31]. Regulation of gene transcription by SETX may contribute to maintaining genome stability by mitigating TRCs, both at promoters [32] and at termination sites of select genes [30, 33]. In addition, SETX is also involved in the repair of DSBs [24, 34–36]. Considering the growing evidence linking Myc-induced RS to TRCs and the role of R-loops in oncogene-induced genomic instability, we set out to address the role of SETX in preventing Myc-induced RS. Here, we show that silencing of SETX induces strong antiproliferative responses associated with cell death and robust activation of the DDR. Mechanistic analysis and genome-wide mapping revealed that this rampant DDR was linked to the accumulation of DSBs proximal to the promoters of genes mapping near early replicated regions. Our findings highlight the requirement for efficient R-loop resolution by SETX at genes prone to undergo TRCs at the early stages of DNA replication and indicate that SETX is a potential therapeutic target in cancer.

## Results

### Loss of Senataxin impairs cell growth upon Myc activation

**Fig. 1:**
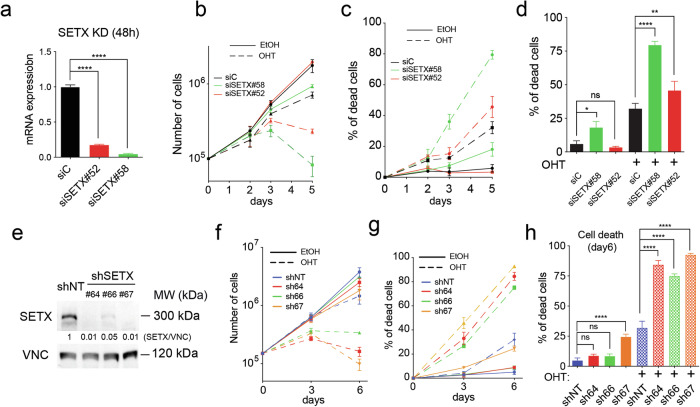
Loss of SETX is synthetic lethal with MycER activation. **a**–**c** 130000 U2OS-MycER cells were transiently transfected with siRNAs against SETX (#52, #58) or a non-targeting siRNA (siC). 400 nM OHT was used to activate MycER. **a** Barplot of the mRNA levels of SETX, normalized on the RPLPO housekeeping gene and expressed as fold change relative to a non-targeting siRNA (siC). Expression analyses were performed 48 h after the transfection in sub-confluent conditions. One-way ANOVA with the Holm-Sidak multiple comparison test was applied to evaluate the statistical significance: **** *p*-value < 0.0001. **b** cell growth curve (**c**) cell death curve of U2OS-MycER cells transfected with the indicated siRNAs. Data points are the average of three replicates (*n* = 3), and error bars are the Stdv. Statistical analyses were performed by Two-way ANOVA with Dunnett’s multiple comparison test: **** *p*-value < 0.0001, *n* = 3. **d** Bar graph reporting cell death (Trypan Blue-positive cells) after five days of culture, an average of three independent replicates. Statistical analyses were performed by One-way ANOVA with Turkey’s multiple comparison test: ns not significant, * *p*-value < 0.05, ** *p*-value < 0.01, **** *p*-value < 0.0001. **d–f** U2OS-MycER cells transduced with shRNAs targeting SETX (#64, #66 and #67) or a non-targeting shRNA (shNT). Silencing was induced with 100 ng/mL of doxycycline. **e** WB analysis, loading control: vinculin (VNC). **f** Growth curve and (**g**) cell death curve of U2OS-MycER cells infected with the indicated shRNAs. **f**, **g** data points are the average of three replicates (*n* = 3), and error bars are the Stdv. **h** Bar graph reporting cell death (Trypan Blue-positive cells, average of three independent replicates) after six days of MycER activation and SETX-KD. Statistical analyses were performed by One-way ANOVA with Turkey’s multiple comparison test: ns not significant, **** *p*-value < 0.0001.

To evaluate the role of Senataxin (SETX) in Myc-induced replicative stress (RS), we took advantage of the human osteosarcoma-derived U2OS-MycER cell line [37], which constitutively expresses the MycER transgene, encoding the Myc protein fused to the estrogen receptor (ER). The MycER chimera is conditionally activated by 4-hydroxytamoxifen (OHT), which induces the translocation of the chimera into the nucleus and transactivates Myc target genes. Conditional activation of the MycER chimera by OHT ligand models oncogenic activation of Myc, recapitulating broad transcriptional regulation of Myc target genes and activation of Myc-induced tumor suppressive responses, including p53 activation, enhanced DNA Damage Response (DDR) and replicative stress (RS) [37]. We conducted loss of function experiments in U2OS-MycER cells by using short-interfering RNAs (siRNAs) against SETX (#52, #58) or a non-targeting siRNA as negative control (siC). siSETXs yielded an 80–95% reduction in SETX mRNA level, as indicated by the RT-qPCR analysis of samples collected at 48 h post-transfection (Fig. 1a). SETX knock-down (KD) had a negligible effect on cell growth at shorter time points (2–3 days) and was tolerated up to 5 days (Fig. 1b and supplementary fig. 1a). The activation of MycER was mildly detrimental to cell proliferation (Fig. 1b and supplementary fig. 1a), as expected, due to Myc intrinsic apoptosis [38]. Of note, MycER activation and the simultaneous silencing of SETX significantly impaired cell growth, thus suggesting that SETX is required for supporting Myc-induced proliferation (Fig. 1b and supplementary fig. 1a). We noticed a substantial increase in the fraction of dead cells when SETX was silenced, and MycER was activated, while silencing of SETX alone had little effects (Fig. 1c, d and supplementary fig. 1b), thus suggesting that SETX may support cell survival and prevent death in cells with oncogenic levels of Myc.

Next, we verified this phenotype with an independent silencing strategy based on conditional short-hairpin RNAs (shRNAs). We transduced the U2OS-MycER cells with lentiviral vectors carrying three different shRNAs targeting SETX (shSETX #64, #66, #67) or, as a negative control, a non-targeting shRNA (shNT). Stably transduced cells were treated with doxycycline to induce the shRNAs and thus to down-modulate SETX expression. RT-qPCR analysis after 72 h of silencing showed a 75% reduction in SETX mRNA levels with all the shRNAs targeting SETX (Supplementary fig. 1c). Silencing was also confirmed at the protein level by western blot analysis (Fig. 1e). Consistently with the results obtained with siRNA-mediated knock-down, silencing of SETX by shRNAs had a mild effect on the growth of cells with physiological levels of Myc, while the combination of MycER activation and SETX KD significantly impaired cell growth at day three and more dramatically at day 6 (Fig. 1f and supplementary fig. 1d). The combined KD of SETX and activation of Myc was associated with increased cell death (Fig. 1g, h and supplementary fig. 1e), thus suggesting that SETX plays an essential function in guaranteeing cell survival and continuous growth in cells with oncogenic levels of Myc. Overall, these results indicate that SETX silencing is synthetic lethal with Myc over-activation and potentially suggests a pivotal role of SETX in regulating Myc oncogenic activity.

### SETX silencing leads to a mild alteration of the cell cycle

**Fig. 2:**
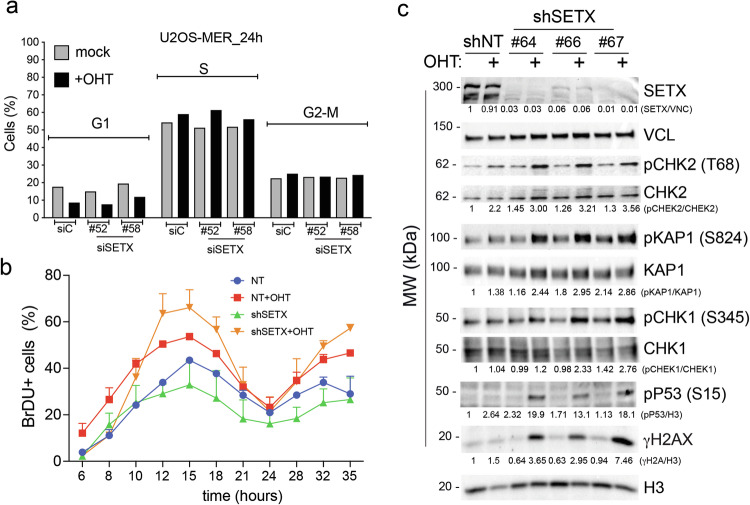
SETX prevents the Myc-induced DNA damage response. **a** Bar graph of cell cycle analysis by FACS of asynchronous U2OS-MycER collected after 24 h of transfection with the indicated siRNAs. OHT was used to activate MycER. **b** FACS analysis of cell cycle progression upon synchronized mitotic exit. Cells were pulse-labeled with BrdU 15 min before collection. Data points are the average of three replicates (*n* = 3), for SEXT silencing each replicate was silenced with a different shRNA. Error bars are the Stdv. **c** WB analysis of DDR markers in U2OS-MycER cells carrying shRNAs against SETX (#64, #66, #67) or a non-targeting sequence (shNT) after 72 h of doxycycline treatment to induce shRNA expression and OHT to activate MycER (Ethanol was used as mock treatment). Vinculin (VNC) and histone H3 (H3) were used as housekeeping genes for loading control.

Given the marked impairment of cell growth and the overt cell death in cells with SETX KD and MycER, we asked whether these phenotypes were also associated with cell cycle alterations. Evaluation of the cell cycle shortly after SETX silencing did not show any relevant effect of SETX silencing on cell cycle distribution in either reference cells or upon MycER activation (Fig. 2a and supplementary fig. 2, upper panel). We next evaluated whether SETX silencing would affect cell cycle progression. We synchronized U2OS-MycER-shSETX cells in G2-M by Nocodazole block, then we released mitotic cells and monitored their synchronous progression from G1 to early and late S-phase (reached respectively 12 h and 18 h post-Nocodazole-release), as well as their re-entry into a second cycle of division (from 24 to 35 h post-Nocodazole-release) (Fig. 2b and supplementary fig. 2, lower panel). Silencing of SETX had no significant impact on S-phase entry and cell cycle progression (Fig. 2b and supplementary fig. 2, lower panel). MycER activation anticipated S-phase entry, both at the first and second cell cycle, as previously reported [8]. This was observed both in shNT and shSETX cells, the latter showing a moderate increase in the fraction of BrdU+ cells, suggesting a further acceleration in S-phase entry (Fig. 2b and supplementary fig. 2, lower panel).

### SETX silencing triggers DDR activation in cells with oncogenic levels of MYC

To assess whether synthetic lethality (SL) may be linked to increased DDR response, we checked the accumulation of DNA damage markers in U2OS-MycER-shSETX cells. Western blot analysis revealed that the simultaneous silencing of SETX and over-activation of Myc triggered phosphorylation of H2A.X (γH2A.X), a marker of the DDR (Fig. 2c), thus suggesting a protective role of SETX in preventing DNA damage upon Myc over-activation. This was confirmed when the analysis was extended to other DDR markers. In particular, Myc activation in shSETX cells increased the phosphorylation of KAP1, Chk2, and p53, which are downstream effectors of the ATM signaling pathway, and also increased pChk1, which is phosphorylated by the ATR kinase (Fig. 2c). This indicates a robust engagement of multiple DDR pathways upon SETX silencing.

### ATR inhibition exacerbates Myc-induced RS in SETX-depleted cells

**Fig. 3:**
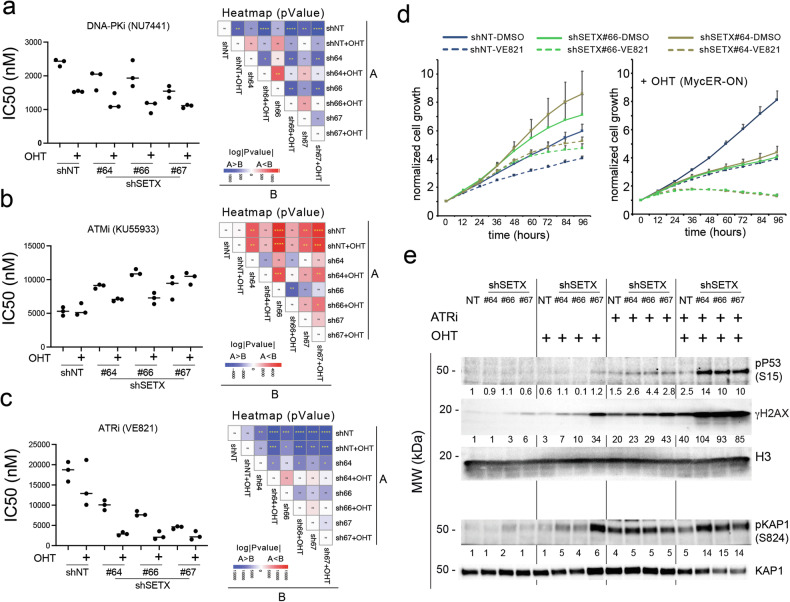
Pharmacological dissection of the role of apical DDR kinases in Myc-induced replicative stress. Pharmacological treatment of U2OS-MycER cells carrying shSETX (#64, #66, #67) or a non-targeting shRNA (shNT). **a–c** Cells were seeded in 96-well plates in three technical replicates in the presence of doxycycline to induce the expression of the shRNAs and of OHT to activate MycER (Ethanol was used as a mock treatment). Seventy-two hours after the treatment, viable cells were analyzed through Cell Titer-Glo Luminescent Cell Viability Assay and IC50 was calculated. *n* = 3. **a** Left, IC50 values for DNA-PKi (NU7441), (**b**) Left, IC50 values for ATMi (KU55933), **c** Left, IC50 values for ATRi (VE821). **a**–**c** Right, heatmaps reporting the *p*-values for the indicated comparison (t-Test, unpaired) color coded in red if the IC50 increased (relative to the shNT) or in blue if the IC50 decreased. *P*-values: ****<0.00001, ***<0.0001, **<0.001 and *<0.01. **d** Growth curves of cells treated with either three µM ATRi or DMSO (mock). **e** WB analysis of cells treated with 3 µM ATRi (VE821) for 48 h. The intensities of the signals normalized to the loading normalizer (H3, GAPDH or KAP1) are reported below each cropped WB scan.

In higher eukaryotes, DNA damage is apically sensed by three main kinases, which activate different DDR pathways: ATM, ATR, and DNA-PK. These play specific functions in response to distinct types of DNA lesions [39]. Given the marked engagement of DDR observed upon MycER activation and SETX silencing, we asked whether these upstream kinases may contribute to the Myc-induced DDR exacerbated by SETX loss. Thus, we assessed the sensitivity of U2OS-MycER-shSETX cells to inhibitors of ATM (ATMi, KU55933), ATR (ATRi, VE821), and DNA-PK (DNA-PKi, NU7441). We determined the half-maximal inhibitory concentration (IC50) in reference cells (U2OS-MycER-shNT) for each compound upon SETX KD, Myc over-activation, or both perturbations. SETX silencing increased cell sensitivity to the DNA-PKi (Fig. 3a). An analogous sensitization was observed in cells with oncogenic levels of Myc, which was only moderately enhanced upon the simultaneous silencing of SETX (Fig. 3a). Thus, DNA-PK activity seemed to have a marginal contribution to the cytotoxic DDR driven by Myc in SETX depleted cells. Myc over-activation did not affect the sensitivity of the cells to ATM inhibition by KU55933, as revealed by the similar IC50 values of shNT cells unstimulated or upon MycER activation (Fig. 3b), thus confirming that ATM does not play a prominent role in Myc induced DDR [5]. Unexpectedly, SETX silencing led to resistance to ATMi, as indicated by the increased IC50 values, possibly implying that loss of SETX is epistatic over ATM inhibition. IC50 values were also higher in cells with MycER activation and SETX KD compared to shNT cells (Fig. 3b), yet lower than those determined in shSETX cells, possibly due to complex interactions between Myc action, silencing of SETX and ATM inhibition. This notwithstanding, these data indicate that the resistance due to SETX KD prevails and that ATMi does not further exacerbate the cytotoxicity of SETX silencing upon Myc activation. VE821 showed remarkable cooperativity with SETX silencing compared to the other inhibitors, indicated by the decreased IC50s determined in silenced cells. IC50s were further reduced by MycER activation (Fig. 3c,d), thus suggesting a protective role of ATR in preventing RS upon SETX loss. Coherently, ATR inhibition triggered a mild DDR response in shSETX cells, which was further increased by MycER activation (Fig. 3e). These data suggest that ATR may protect cells from loss of SETX and imply that SETX may be required to safeguard DNA replication and curb Myc-induced RS [40].

### SETX mitigates Myc-induced RS

**Fig. 4:**
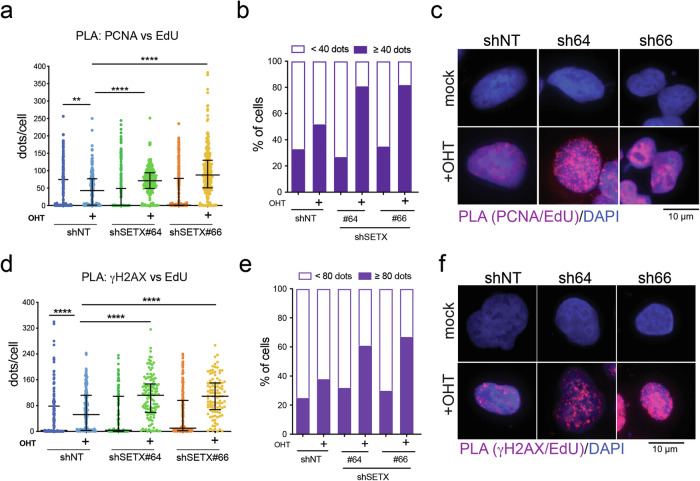
SETX restrains Myc-induced replicative stress. U2OS-MycER cells carrying shRNAs against SETX (#64, #66) or a nontargeting shRNA (shNT) were seeded in the presence of doxycycline to induce the expression of the shRNA and of OHT to activate MycER. Ethanol was used as mock treatment. After 48 h, cells were synchronized at prometaphase by 100 ng/mL of Nocodazole for 8 h. Synchronized cells were released and collected after 18 h (late S phase). Before fixation, cells were pulsed with 25 µM of EdU to label the newly synthesized DNA. **a**–**c** Replication foci were measured by PLA to assess the proximity between nascent DNA and the replisome (PCNA). Sample size: 207–275 cells per condition. Kruskal Wallis test with Dunn’s multiple comparison correction was applied to run statistical analysis: ** *p*-value < 0,01, **** *p*-value < 0,0001. **d**–**f** Analysis of proximity of DDR foci (γH2AX) and newly synthesized DNA (EdU-labeled DNA) by PLA. Sample size: 102–257 cells per condition. Kruskal Wallis test with Dunn’s multiple comparison correction was applied to run statistical analysis: **** *p*-value < 0,0001. **b**, **e** Bar graphs show the fraction of cells in each condition with the indicated number of PLA dots. **c**, **f** Representative micrographs of PLA-foci. In blue, the nuclei stained with DAPI; in red, the PLA signal.

Several reports have linked Myc synthetic lethal interactions to genes whose activity prevents replicative stress [7, 41]. Considering this and the results shown so far, we probed whether loss of SETX would modulate Myc-induced RS. First, we assessed replication foci by measuring the proximity of PCNA and newly synthesized DNA by proximity ligation assay (PLA). To enrich the population of replicating cells, U2OS-MycER-shSETX cells were synchronized in the G2-M phase, pulsed with EdU (2 h), and collected in late S-phase (18 h post-Nocodazole-release). Foci due to the co-localization of PCNA and nascent DNA were increased by MycER activation (Fig. 4a-c and supplementary fig. 3), as expected due to the compensatory increase in origin firing upon Myc-induced RS. The number of foci was further enhanced by silencing SETX on top of MycER activation, while silencing of SETX alone did not significantly alter the number of foci detected (Fig. 4a-c). Since the prolonged stalling of replication forks can lead to fork collapse, which is associated with the generation of DSBs, we also assessed whether the increased DDR upon Myc activation was associated with newly replicated DNA. Indeed, silencing of SETX upon Myc-activation further increased the detection of proximity between γH2A.X and EdU-labeled DNA, while no increase was detected upon silencing of SETX (Fig. 4d-f and supplementary fig. 4). Overall, these findings indicate that loss of SETX increases Myc-induced RS.

### SETX prevents transcription-replication conflicts (TRCs)

**Fig. 5:**
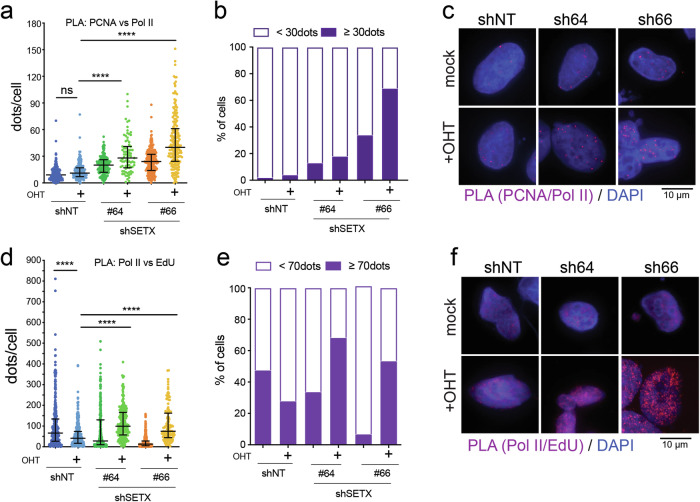
Myc over-activation exacerbates TRCs upon SETX-KD. U2OS-MycER cells carrying different shRNAs against SETX (#64, #66, #67) or a non-targeting sequence as negative control (shNT) were seeded in 150 cm plates in the presence of doxycycline to induce the expression of the shRNA and OHT to over-activate Myc. Ethanol was used as a mock treatment. After 48 h of treatment, cells were synchronized in prometaphase by 100 ng/mL Nocodazole for 8 h. Synchronized cells were released for 18 h and then pulsed with 25 µM of EdU before collection. TR-conflicts were measured by PLA assay to assess the proximity between (**a**–**c**) RNA pol II and PCNA protein or (**d–f**) RNA pol II and newly synthesized DNA (EdU labeled). **a**, **d** Dot plot showing the quantification of the PLA foci per cell. Sample size: 79–270 cells per condition (**a**), 114–462 cells per condition (**d**). Kruskal Wallis test with Dunn’s multiple comparison correction was applied to run statistical analysis: ns not significant, **** *p*-value < 0,0001. **b**, **e** Bar graphs showing the fraction of cells with the indicated number of PLA dots for each condition. **c, f** Representative micrographs of PLA-foci. In blue are the nuclei stained with DAPI; in red are the PLA signals.

Given that SETX is one of the helicases involved in R-loop resolution and considering that R-loops are a potential source of genomic instability due to collision between the DNA replication machinery and the transcription apparatus, we assessed TRCs by PLA on RNA pol II (to probe transcription) and PCNA (to probe DNA synthesis). Myc over-activation did not significantly increase PLA signals compared to untreated cells (Fig. 5a–c and supplementary fig. 5), implying Myc may restrain TR-conflicts to support its oncogenic activity. Loss of SETX significantly raised RNA pol II-PCNA proximity (Fig. 5a-c and supplementary fig. 5), thus indicating an inherent function of SETX in preventing TRCs. Notably, activation of MycER combined with SETX KD further enhanced the number of foci detected (Fig. 5a–c and supplementary fig. 5), suggesting a higher probability of TR-encounters in these conditions. Similarly, PLA assay to assess the proximity of RNA Pol II to nascent DNA revealed an increase of TRCs (i.e., RNA Pol II and EdU foci) in cells with simultaneous activation of Myc and SETX KD (Fig. 5d–f and supplementary fig. 6). Overall, these results indicate that SETX prevents TRCs, which are further exacerbated in the context of Myc oncogenic activation.

### SETX silencing leads to the accumulation of R-loops in cells with high Myc activity

**Fig. 6:**
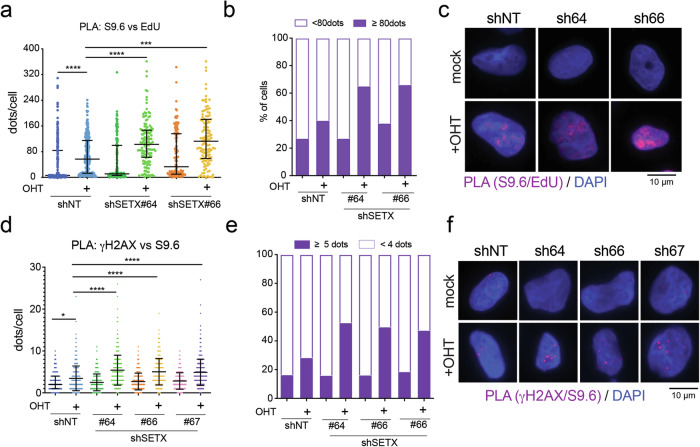
SETX limits Myc-driven R-loops accumulation near newly synthesized DNA DRR foci. U2OS-MycER cells carrying different shRNAs against SETX (#64, #66, #67) or a non-targeting sequence as negative control (shNT) were seeded in the presence of doxycycline to induce the expression of the shRNA and OHT to activate MycER. Ethanol was used as a mock treatment. After 48 h, cells were synchronized in prometaphase by 100 ng/mL Nocodazole for 8 h. Synchronized cells were released for 18 h and pulsed with 25 µM of EdU before collection. Fixed cells were stained for PLA to measure the proximity of RNA-DNA hybrids (S9.6) to nascent DNA (EdU labeled DNA) (**a**–**c**) or to DDR foci (ɣH2Ax) (**d–f**). **a, d** Dot plot showing the quantification of the PLA foci per cell. Sample size: 120–288 cells per condition (**a**), 400–600 cells per condition (**d**). Kruskal Wallis test with Dunn’s multiple comparison correction was applied to run statistical analysis: *p-value < 0,05, *** *p*-value < 0,001, **** *p*-value < 0,0001. **b, e** Bar graphs show the fraction of cells in each condition with the indicated number of PLA dots. **c, f** Representative micrographs of PLA-foci. Blue: DAPI stained nuclei, red: the PLA signals.

Given the role of SETX in resolving R-loops and preventing TRCs, and considering the potential role of TRCs as a cause of RS [17], we then asked whether loss of SETX would lead to the accumulation of R-loops proximal to newly synthesized DNA. To address this, we performed PLA between R-loops, detected by the S9.6 antibody, and nascent DNA labeled by EdU. MycER activation enhanced the proximity between R-loops and the neo-synthesized DNA (Fig. 6 a-c and supplementary fig. 7), indicating that Myc-driven R-loops are generated near active replication forks. This coherently fits with the evidence that over-activation of Myc triggers premature replication of G1 transcribed regions, predisposing the cells to TRCs [8]. While SETX silencing alone mildly increased R-loop proximity to nascent DNA, depletion of SETX in MycER-activated cells strongly enhanced these encounters (Fig. 6a–c and supplementary fig. 7), thus suggesting that SETX may limit Myc-driven R-loop accumulation close to early replicated regions. To evaluate whether the persistence of R-loops may be linked to increased fork collapse and generation of DSBs, we assessed whether ɣH2Ax foci may colocalize with R-loops. PLA assay revealed a moderate and reproducible increase of ɣH2Ax-R-loop foci (Fig. 6d–f and supplementary fig. 8), suggesting that this increased incidence of R-loops proximal to newly synthesized DNA may trigger genomic instability in MycER cells. Overall, these data indicate that loss of SETX leads to a higher frequency of TRCs and DSBs in cells with elevated Myc activity.

### Silencing of SETX exacerbates DSBs at TRCs in early replicated regions

**Fig. 7:**
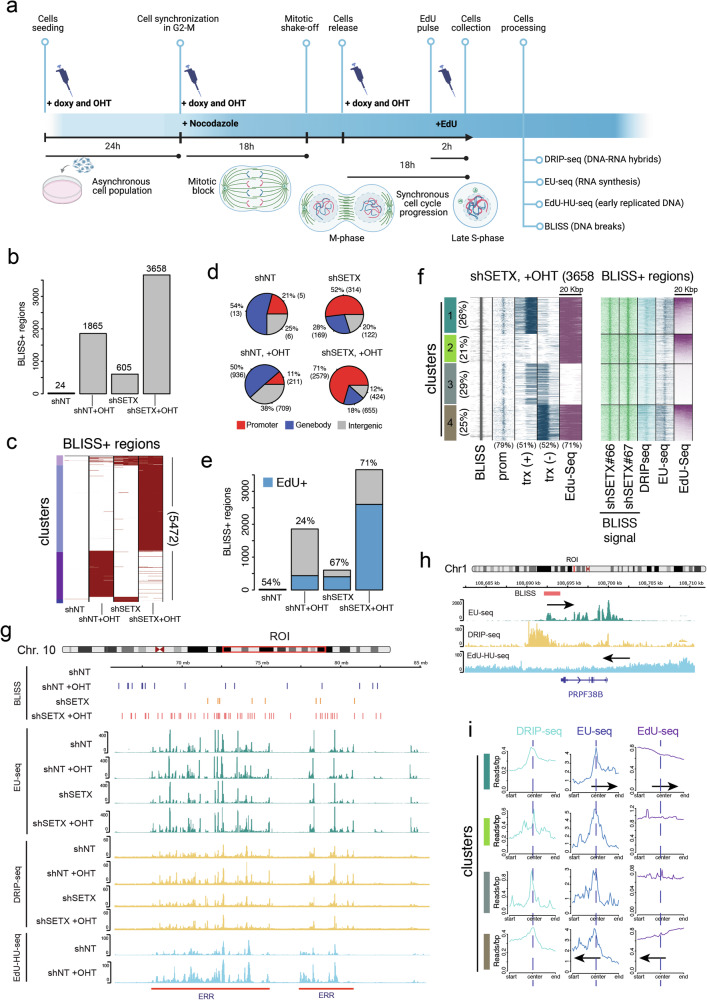
Genome-wide analyses and mapping of DSBs. Synchronized U2OS-MycER cells carrying shSETX (with either sh66 or sh67) or shNT (non-targeting control) were sampled during S-phase and analyzed. **a** scheme of the experimental design and genomic analyses (**b**) BLISS+ regions identified upon SETX silencing and MycER activation ( + OHT). Created in BioRender. Campaner, S. (2025) https://BioRender.com/g85c195. (**c**) Clustered Heatmap reporting the overlap of the BLISS+ regions. **d** Pie-chart of the genomic annotation of BLISS+ regions. **e** Histogram of the BLISS+ regions with their overlap with early replicated regions (ERRs). The fraction of the BLISS+ regions overlapping with ERRs is reported in blue, and values (in %) are reported above each histogram. (=**f** Left, heatmap of the BLISS+ regions detected in shSETX-MycER cells, centered on the BLISS+ regions and clustered by transcripts and ERRs (detected by EdU-HU-seq). Fraction of BLISS+ intersecting each element are reported below. Right, heatmap of the signals (reads) detected by BLISS (DSBs), DRIP-seq (RNA-DNA hybrids), EU-seq (nascent RNA), and EdU-HU-seq (newly synthesized DNA on ERRs). **g, h** Chromosome level genome browser snapshot. ROI is the region of interest displayed. The red bar below highlights the early replicated regions (ERR). Arrows in **h** indicate the direction of gene transcription (from gene annotation) and DNA synthesis (from EdU-HU signals). **i** Metagene profiles of the clusters identified in **f**.

To seek further mechanistic insight into the origin of genomic instability triggered by loss of SETX in MycER cells, we mapped DNA breaks by Breaks Labeling In Situ and Sequencing (BLISS) (Fig. 7a) [42]. We identified BLISS+ regions (i.e., those genomic regions enriched in DSBs), as previously described [14]. In line with previous evidence [14], activation of MycER increased the number of BLISS+ regions (from 24 to 1856, Fig. 7b), confirming that enhanced Myc activity can be associated with genome instability. Also, loss of SETX compromised genomic integrity, although to a lesser extent than activation of MycER (605 vs. 1856 BLISS+ regions detected, respectively) (Fig. 7b). Combined MycER activation and SETX silencing led to a further increase in BLISS+ regions (3658 BLISS+ regions) to levels exceeding those detected in either MycER or shSETX cells (Fig. 7b). Of note, BLISS+ regions in shSETX-MycER cells showed a negligible overlap with those detected upon MycER activation (Fig. 7c and supplementary fig. 9d), and a partial overlap with those detected in cells silenced for SETX (11% of the BLISS+ regions in shSETX-MycER cells were also detected in shSETX cells) (Fig. 7c and supplementary fig. 9c). This indicates that the genomic instability observed upon MycER activation in shSETX cells was not additive but more likely resulting from a synergistic effect of the two perturbations: Myc activation and SETX silencing. BLISS+ regions detected in either mock or MycER-activated cells were predominantly evenly distributed between intragenic and extragenic regions, with a minor fraction localized at promoters (Fig. 7d). Instead, upon loss of SETX expression or its loss combined with MycER activation, we detected BLISS+ regions predominantly mapping at promoters (52% and 71%, respectively) (Fig. 7d).

Considering that genomic instability triggered by elevated Myc has been linked to the transcription of genomic regions that are replicated early during S-phase [8, 14], we next evaluated whether the BLISS+ regions in our analysis might be positioned near early replicated regions (ERRs). To this end, we utilized a previously published map of ERRs determined by EdU-HU-seq analysis of U2OS cells [14]. Clustering of BLISS+ regions based on their overlap with ERRs and S-phase transcripts (from EU-seq) revealed that DSBs triggered by MycER activation were only moderately associated with either early replicated regions (24% of BLISS+ regions overlapped with an ERR) (Fig. 7e and supplementary fig. 10) or genes transcribed in S-phase (36% of BLISS+ regions overlapped with a transcribed gene and supplementary fig. 10). Instead, DSBs arising in shSETX cells, in addition to being enriched in transcribed genes (supplementary fig. 11), also showed a more pronounced occurrence at ERRs (63% were proximal to an ERR) (Fig. 7e and supplementary fig. 11). Furthermore, analyses of BLISS+ regions detected upon MycER activation in shSETX cells revealed that DSBs were close to the promoters of S-phase transcribed genes (79%, Fig. 7f, clusters 1, 3 and 4) and frequently associated with early replicated regions (71%, Fig. 7e, f, clusters 1, 2 and 4), compatible with the hypothesis that these DSBs are a consequence of TRCs. This was confirmed by the analysis of signal distributions along the genome, which showed the clustering of BLISS+ regions detected upon SETX silencing and MycER activation in the proximity of ERRs and S-phase transcribed genes (Fig. 7g,h and supplementary fig. 12). Directionality analysis of transcription (based on promoter annotation and EU-signals) and DNA replication (based on the pattern of EdU-HU-seq signals) revealed that DSBs were frequently positioned where newly synthetized DNA from ERRs would encounter S-phase transcribed genes, either co-directional or in a head-on configuration (i.e., genes transcribed in the same direction of replicated DNA or in the opposite direction, respectively) (Fig. 7i); with cluster 1 and 4 showing a prevalence of co-directionality.

### SETX is not essential for the processing of co-transcriptional R-loops

Given that SETX can resolve R-loops and that R-loops have been shown to be potential causal factors that may promote genomic instability, we then mapped R-loops genome-wide by spiked-in DRIP-seq [43]. Only a limited number of transcribed loci showed a statistically significant difference in DRIP-seq signals upon SETX silencing, mainly in cells with combined activation of MycER, with a preponderance of genes showing a reduction of DRIP-seq signals (supplementary fig. 13). Of note, DRIP-seq signals were highly correlated with nascent RNA, thus suggesting that the level of the RNA-DNA hybrids depended more strongly on transcription rates rather than their downstream processing (supplementary fig. 13). Loci with either increased or decreased DRIP-seq signals did not show a strong association with BLISS+ regions (supplementary fig. 14), thus indicating that such alterations in RNA-DNA hybrids may not be causal in exacerbating Myc-induced genomic instability (supplementary fig. 14). Moreover, BLISS+ regions detected upon MycER activation and SETX silencing did not show relevant alterations (either increase or decrease) of DRIP-seq signals when MycER was activated, or SETX was silenced (supplementary fig. 15), thus indicating that loss of SETX did not lead to recurrent and consistent accumulation of R-loops at sites prone to DSBs formation upon Myc activation. These observations suggest that loss of SETX might not alter the processing of co-transcriptional RNA-DNA hybrids (more stable and detectable at a population-wide level by DRIP-seq) but may instead be necessary to process transient R-loops at TRC-prone regions. Given the stochastic nature of such events, the transient accumulation of RNA-DNA hybrids at these loci may escape detection by DRIP-seq.

## Discussion

Replicative stress represents an attractive therapeutic opportunity to tackle Myc-induced tumors, given that these tumors are prone to undergo cytotoxic DNA damage once RS is exacerbated [7, 40, 41]. Here, we have shown that loss of SETX can be exploited to trigger cell death and block cell growth in cells with elevated Myc activity. This was associated with rampant RS, leading to a robust activation of the DNA damage response, engaging both the ATM and the ATR signaling. Enhanced replicative stress was accompanied by increased transcription-replication conflicts (TRCs) and the accumulation of R-loops proximal to newly synthesized DNA and DDR foci.

On the other hand, genome-wide mapping of R-loops by DRIP-seq failed to detect widespread accumulation of R-loops at transcribed loci upon SETX silencing, neither in unchallenged cells nor upon MycER activation. This suggests that SETX may not be essential for the resolution of co-transcriptional RNA-DNA hybrids but is rather selectively involved in the resolution of R-loops associated with TRCs in order to avoid the collapse of the replicative fork undergoing RS. Of note, these events are intrinsically stochastic and elusive to detection by population-wide analyses, like, for instance, DRIP-seq (see below for further discussion on this point). This may explain why single-cell quantitative analysis of R-loops by PLA detected an increase in R-loops associated with TRCs and DDR foci, while DRIP-seq failed to do so. Coherently with the PLA data, genome-wide mapping of DSBs arising upon SETX silencing and MycER activation indicated a bias for their localization at TRCs near early replicated regions.

Increased DNA damage upon SETX silencing is in line with previous evidence linking loss of function or silencing of SETX to enhanced DNA damage and genomic instability, frequently related to TRCs generated by persistent R-loops [20, 23, 44, 45]. For instance, SETX prevented DNA damage by favoring faithful termination of transcription [30, 33] and by resolving R-loops at promoter-proximal regions [46]. Thus, in principle, loss of SETX may predispose to alterations in R-loops at either promoter-proximal regions or at termination sites, potentially leading to DSBs at these sites once transcription-replication conflicts ensue. Considering that the DSBs we detected in SETX KD cells upon MycER activation are mainly proximal to promoters, it is reasonable to exclude defective termination of transcription as a causal factor of the genomic instability we observed. On the other hand, given we failed to detect a consistent increase in promoter-proximal R-loops associated to DSBs we deem unlikely that DNA breaks may be resulting from the constitutive accumulation of RNA-DNA hybrids at transcribed genes, since if this were the case, we should have been able to document increased DRIP-seq signals at least at those sites where DSBs were detected. This reasoning leads to alternative explanations accounting for the genotoxic effect of the loss of SETX, in line with recent data suggesting additional roles of SETX in managing R-loops implicated in TRCs. Indeed, SETX can physically associate and travel with the replisome to stabilize the replicative fork and support processive DNA synthesis by resolving those R-loops that are encountered during DNA replication in transcribed regions [44, 45, 47]. A separation of function SETX mutant, whose association to the replisome was compromised, demonstrated that replisome association was sufficient to preventing genomic instability [47]. Interestingly, this defective SETX mutant also failed to clear promoter-paused RNA-pol2 at TRC sites [47]. This model may explain (i) the stochastic nature of R-loops that are resolved by SETX in MycER cells (e.g. not all the R-loops are resolved by SETX, but only those that are stochastically engaged at TRCs) and (ii) the prevalence of promoter-associated DSBs we detected when SETX was silenced in MycER cells (these will be the consequence of the persistence of R-loops at TRCs). In line with our observations, this SETX mutant per se was not sufficient to trigger a genotoxicity, but it required an additional event to boost TRCs and genomic instability [47], which in our experiments was provided by the activation of Myc.

In addition, since SETX is recruited to DSBs on active genes and prevents illegitimate repair with distant loci [48, 49], its loss may also exacerbate DNA damage by compromising the faithful resolution of DSBs occurring at TRC sites triggered by Myc.

Based on the above reasoning, we propose that genomic instability caused by loss of SETX in Myc overexpressing cells is primarily due to a specific role of SETX in resolving R-loops encountered by the replisome and may be further exacerbated by defective DNA repair.

Generation of DSBs engages the DDR, which may either be protective (pro-survival) or contribute to the demise of damaged cells. In our experiments, we detected activation of both the ATR and the ATM pathways. To assess the role of the DDR engaged by SETX silencing, we performed a loss of function analysis using pharmacological inhibitors of ATR, ATM, and DNA-PK, the main apical kinases involved in the DDR. ATR inhibition had a strong synergy with SETX silencing both in mock and upon MycER activation. This could be due to the fact that ATR, by controlling the replication checkpoint, may limit fork progression and, therefore, prevent TRCs [50]; consequently, inhibition of ATR will enhance TRCs and thus exacerbate DDR upon SETX silencing. ATR is also required for the recovery of replicative forks stalled at co-transcriptional R-loops; this is mediated by the engagement of MUS141-SLX4-RAD52-POLD3 [17]. In this scenario, enhanced toxicity of ATR inhibition may be explained by the reduction of fork restart at TRCs caused by SETX loss. On the other hand, the toxicity of ATM inhibition was alleviated by SETX silencing, possibly suggesting that the reduced cell fitness triggered by the loss of SETX might in part be due to ATM activity, while inhibition of DNA-PK did neither sensitized nor induced resistance to SETX silencing.

SETX has been proposed as one of the helicases able to resolve co-transcriptional loops, yet in our analyses, upon SETX silencing, we detected mild changes in R-loops only in a restricted number of loci, and we did not see a widespread increase in R-loops at transcribed loci by performing DRIP-seq. Instead, we more consistently observed a loss of DRIP-seq signals that were associated with the downregulation of nascent RNA. This lack of a widespread increase of R-loops upon SETX silencing might indicate the redundancy of SETX with other helicases in controlling co-transcriptional R-loops or the inability of DRIP-seq to detect subtle changes in R-loop dynamics due to altered RNA processing at transcribed loci. The latter hypothesis is supported by the strong correlation of DRIP-seq signals and nascent RNA we observed, which suggests that the major contribution to DRIP-seq signals is due to RNA synthesis rather than the processing of RNA-DNA hybrids. Either way, our findings are in line with recent reports, where the loss of SETX did not result in gain but rather in loss of DRIP-seq signal on coding genes [51, 52]. In support of a redundant/nonessential role of SETX at R-loops on protein-coding genes, we also note that while previous work detected SETX at DSBs, the same report failed to detect SETX at transcribed genes despite the presence of RNA-DNA hybrids at these loci [48], thus suggesting that either SETX is recruited very transiently to transcribed genes or that other helicases are processing R-loops at these loci. In conclusion, we propose that SETX prevents TRCs upon Myc activation by resolving R-loops that encounter the replisome.

We should point out that our study, utilizing isogenic cellular models, primarily focused on elucidating the underlying mechanisms. This highlights the need for future studies to validate these findings in clinically relevant settings. Notably, in-silico analysis using the DepMap database [53] supports the concept of synthetic lethality between SETX loss and Myc overexpression in certain tumors (Supplementary Fig. 16a). Furthermore, a pan-cancer analysis of the TCGA data [54, 55] revealed that while Myc amplification is associated with poor prognosis, the presence of SETX missense mutations is linked to improved outcomes in Myc-amplified tumors (Supplementary Fig. 16b, c). On the other hand, mutation of SETX in tumors without Myc amplification did not affect disease progression (Supplementary Fig. 16b, c). This finding support that the loss of SETX function may mitigate the oncogenic activity of MYC.

While our study and others have implicated SETX as a key factor in supporting cancer cell fitness, suggesting its potential as a therapeutic target, it’s crucial to acknowledge the evidence that SETX can also act as a tumor suppressor in specific contexts [23, 29]. Future preclinical efforts should carefully consider this dual role. Further research is needed to determine how exploiting the vulnerability associated with SETX loss-of-function can be leveraged to enhance the efficacy of cytotoxic therapies targeting Myc-driven cancers. This could pave the way for novel single-agent or combinatorial treatment strategies that exploit replicative stress.

## Materials & Methods

### Cell lines and culture condition

U2OS-MycER cells [37] and HEK-293T cells were grown in 90% DMEM (Dulbecco’s Modified Eagle’s Medium) supplemented with 10% FBS (South-America origin), L-Glutamine (2 mM) and 1% Penicillin-Streptomycin, in adhesion at 37 °C with 5% CO2. U2OS-MycER-shSETX cells were grown in the same culture conditions as the parental cell line, except that the medium was supplemented with tetracycline-free FBS, in order to avoid the spurious expression of the short hairpin RNA (shRNA), and with 1 μg/mL of Puromycin. The MycER transgene was activated with 400 nM of 4-hydroxytamoxifen (OHT), while shRNAs were induced with 100 ng/mL of doxycycline. To generate a synchronous cell population, 3×10^6^ cells were seeded in 150 cm plates and grown for 48 h before being treated with 50–100 ng/mL of Nocodazole (Sigma, Cat. No. SML-1665) for 8 h to block cells in mitosis. Mitotic cells were retrieved by shaking the plates off, collected at 1000 rounds per minute (rpm) for 5 min at room temperature and washed twice with Phosphate Buffered Saline (PBS). Cells reseeded for synchronous cell cycle progression, collected at different time points from the release and fixed either in 70% ice-cold Ethanol (diluted in H_2_O) while vortexing (for FACS analysis), or in 3% Formaldehyde/ 2% Sucrose (diluted in PBS) for 10 min at room temperature (for proximity ligation assay, PLA). Cell lines were provided as mycoplasma-free by the IEO-tissue Unit (https://www.research.ieo.it/research-and-technology/technological-units/cell-culture-unit-ccu/).

### Cells transfection with siRNAs

For the reverse transfection with short interfering RNAs (siRNAs) in 6 well plates, 3.25 µL of lipofectamine RNAiMAX reagent (Lifetech) were firstly diluted in 500 µL of Opti-MEM (Gibco) and then incubated for 15 min at room temperature with 12.5 nM of siRNAs, previously diluted in 500 µL of Opti-MEM, in order to obtain a 1 mL transfection mix for each well. Cells were resuspended in medium with 2x FBS and without antibiotics, at a final concentration of 130,000 cells/mL. 1 mL of cell suspension was seeded in each well on top of the 1 mL of transfection mix. For the reverse transfection in 10 cm plates, 15 µL of lipofectamine were diluted in 2 mL of Opti-MEM and 12.5 nM siRNA were diluted in 2 mL of Opti-MEM (4 mL of transfection mix per plate). 800.000 cells were resuspended in 4 mL of medium with 2x FBS and without antibiotics and seeded on top of the transfection mix in a final volume of 8 mL per plate (cells plus transfection mix). The knock-down efficiency was evaluated in sub-confluent conditions after 48–72 h from the transfection.

### Viral production and transduction

The U2OS-MycER cell line was generated by transducing the U2OS cells with retrovirus carrying the MycER chimera [37]. For stable silencing, U2OS-MycER cells were transduced with three different doxycycline-inducible human shRNAs against SETX (ULTRA-3250464-pZIP-TRE3G-ZnGreen-Puro, ULTRA-3250466-pZIP-TRE3G-ZnGreen-Puro, ULTRA-3250467-pZIP-TRE3G-ZnGreen-Puro) purchased from shERWOOD (Transomic). As control, we transduced U2OS-MycER cells with a doxycycline-inducible non-targeting shRNA (TLNSU4300) purchased from shERWOOD (Transomic). To produce the lentiviral particles, HEK-293T cells were plated in 10 cm plates 24 h before transfection and transfected at 80% confluency with Lipofectamine3000 following the manufacturer’s instructions (Invitrogen). Briefly, 500 µL of Opti-MEM (Gibco) supplemented with 15uL of Lipofectamine3000 were mixed 1:1 with 500 µL of Opti-MEM containing the DNA plasmids and 20uL of the p3000 reagent and incubated for 15 min at room temperature. Each 10 cm plate was transfected with 10 µg of the lentiviral plasmid carrying the shRNA, and with helper plasmids (5 µg of VSVG and 6 µg of δ8.2) carrying the viral genes Pol, Env and Gag. The transfection mix (1 mL) was applied dropwise over the cells. After 6 h the cell medium was replaced with fresh one (8 mL per plate). The viral supernatant was collected at 24 h and at 48 h post-wash out, filtered with 0.45 μm filter and stored at −80 °C. For the transduction, 250000 U2OS-MycER cells were seeded in 6 well plates and, after 24 h, the cells were incubated with 2 mL of lentiviral supernatant supplemented with 8 µg/mL of Polybrene. After 8 h from the transduction, the cell medium was replaced with fresh one. 24 h later, the cells were trypsinized and seeded in Puromycin (1 µg/mL)-containing medium for the selection of the infected cells.

### Protein extraction and western blot analysis

For western blot (WB) analysis, sub-confluent cells were lysed in 6 well plates by scraping them on ice with 200 μL of cold lysis buffer (20 mM HEPES, 0.5 M NaCl, 5 mM EDTA, 10% glycerol and 1% Triton X-100) supplemented with MINI-complete Protease Inhibitor and phospho-STOP inhibitor cocktail (Roche). Lysis was carried out for 20 min on ice, then cell lysates were sonicated with 10 cycles of 30 seconds ON/OFF at the maximum power (Bioraptor plus) and cleared by centrifugation at 13000 rpm for 20 min at 4 °C. Extracted proteins were quantified by Bradford assay (Bio-Rad Laboratories). 30 μg of proteins were incubated at 95 °C for 5 min with Laemmli sample buffer (350 mM Tris-HCl pH 6.8, 30% glycerol, 10% SDS, 0.1% bromophenol blue, 6% β-mercaptoethanol) and loaded on 4–15% gradient precast TGXTM polyacrylamide gel (Bio-Rad Laboratories). Proteins were blotted onto a nitrocellulose membrane using the Trans-Blot® Turbo^TM^ transfer system for 30 min at 25 Volt and 1 Ampere. After the Ponceau staining and destaining, the membrane was blocked with 5% (w/v) of Bovine Serum Albumin (BSA) diluted in Tris Buffered Saline-Tween (0,1% v/v) (TBS-T) for 1 hour at room temperature and incubated overnight with the primary antibody at 4 °C. Primary antibodies (listed in Table 2) were diluted 1:1000 in TBS-T containing 5% of BSA. After 3 washes in TBS-T (15 min each), the membrane was incubated for 1 hour at room temperature with the appropriate peroxidase-coupled secondary antibody, diluted 1:10000 in TBS-T. ECL-based chemiluminescence (Bio-Rad Laboratories) was detected through the BioRad ChemiDoc system and images were processed with Image Lab 4.0 (Bio-Rad Laboratories).

### RNA extraction and RT-qPCR

Sub-confluent cells were trypsinized, centrifuged at 1200 rpm for 5 min and washed once in PBS. RNA was extracted by using the Quick-RNA MiniPrep kit (Zymo Research) in accordance to the manufacturer’s instructions. Briefly, cells were lysed by adding 300 µL of RNA lysis buffer to the cell pellet and the lysate was passed through a Spin-Away™ filter to remove genomic DNA. The flowthrough was mixed 1:1 with Ethanol 100% and loaded onto a Zymo-Spin™ IIICG Column to capture RNA. The column was washed with 400 µL of RNA Wash Buffer before proceeding with the DNAse I treatment: 5 µL of DNase I (1 U/µl) were mixed with 75 µL of DNA Digestion Buffer and the mixture was applied directly onto the column matrix. After 15 min of incubation at room temperature, the column was washed once with 400 µL of RNA Prep Buffer and then twice with 700 µL of RNA Wash Buffer. An additional centrifugation at full speed for 1 min at room temperature was performed to eliminate residual buffers. RNA was eluted by adding 30–50 μL of RNase-free water onto the column and centrifuging for 1 minute at full speed. The extracted RNA was quantified using Nanodrop (Thermo Fisher Scientific). Purified RNA was then used for cDNA synthesis with ImPromII Reverse Transcription System (Promega). Briefly, 1 µg of RNA was combined with 1 μL of Oligo-dT and 0.1 μL of random primers diluted in RNase-free water in a final volume of 30.25 μL and incubated for 5 min at 70 °C to eliminate secondary structures. After 5 min on ice, samples were mixed with 19.75 μL of a solution containing 5 μL of 25 mM MgCl2, 10 μL of 5X RT buffer, 2.5 μL of 10 mM dNTP mix, 1.25 μL of RNase inhibitor and 1 μL of Reverse Transcriptase (RT). The mixture was incubated first at 25 °C for 5 min to allow primers annealing, then at 42 °C for 60 min to allow RT elongation, and lastly at 70 °C for 15 min to inactivate the RT enzyme. Synthesized cDNA was used for subsequent Real-time quantitative Polymerase Chain Reaction (RT-qPCR). 10 ng of cDNA were mixed with 10 μL of Sybr Green 2X (Thermo Fisher) and with 500 nM of primers in a final reaction volume of 20 μL. RT-qPCR reactions were performed with BioRad CFX 96 System in three technical replicates.

### Cell cycle analysis by fluorescence-activated cell sorting (FACS)

Synchronized cells were pulsed with 33 μM of 5-bromo-2-deoxyuridine (BrdU) for 30 min prior to being harvested. After one wash in PBS, around 2×10^6 cells were collected by centrifugation at 2000 rpm at 4 °C for 5 min, fixed with 1 mL of dropwise ice-cold 70% Ethanol, while vortexing, and stored overnight at 4 °C in 70% Ethanol. Afterward, cells were washed with 1% BSA in PBS-and resuspended in 2 mL of 2 N HCl denaturing solution for 20 min at room temperature to expose labeled DNA. Then, HCl was neutralized by adding 3 mL of 0.1 M Sodium Borate (Na2B4O7, pH 8.5) for 2 min at room temperature. Cells were washed twice with PBS and resuspended in 100 µL of anti-BrdU antibody (which features are listed in Table 2) diluted 1:5 in PBS 1% BSA for 1 hour at room temperature. After a quick wash in PBS, cells were incubated with 100 µL of Alexa 647-conjugated secondary antibody diluted 1:100 in PBS-1% BSA for 1 hour at room temperature light-protected. Lastly, cells were washed with PBS and resuspended in 500 µL of 2.5 μg/mL of propidium iodide (PI) and 250 μg/mL of RNase A diluted in PBS, overnight at 4 °C. Cell cycle was assessed by MACSQuant flow cytometer and raw data were analyzed through FlowJo software. To check the synchronization of cells before PLA, synchronized cells were pulsed with 25 μM of 5-ethynyl-2-deoxyuridine (EdU) for 2 h prior to being harvested. After one wash in ice-cold PBS, approximately 10^6^ cells were fixed by dropwise addition of 1 mL of ice-cold 90% methanol while vortexing, and stored overnight at 4 °C. Then, the 90% methanol were removed by washing cells with ice-cold PBS and cell permeabilization was performed by resuspending the pellet in 200 µL of 0.2% Triton X-100 diluted in PBS, for 30 min at room temperature. After one wash in 200 µL of ice-cold PBS, cells were incubated for 30 min at room temperature light-protected with the Click-iT reaction cocktail® (Life Technologies), containing 859 µL of 100 mM of Tris-HCl pH 8, 40 µL of 100 mM of copper sulfate (CuSO4), 100 µL of 1 M sodium-ascorbate and 1 µL of Alexa Fluor 647 azide 1 M. After one wash in ice-cold PBS, cells were resuspended in 200 µL of 2.5 μg/mL of PI and 250 μg/mL of RNase A diluted in PBS, and kept overnight at 4 °C. Cell cycle was assessed by MACSQuant flow cytometer and raw data were analyzed with the FlowJo software.

### Proximity ligation assay (PLA)

Proximity Ligation Assay (PLA) was performed by integrating the Duolink® In Situ PLA protocol (Sigma-Aldrich) with the Click-iT® reaction step (Life technologies). Briefly, 1×10^6^ synchronized cells, seeded over cover glasses in 60 cm plates, were pulsed with 25 μM EdU for 2 h prior to being harvested. Cells were permeabilized with 3 mL of 0.5% Triton X-100 (diluted in PBS) for 10 min at 4 °C and then fixed with 3 mL of 3% Formaldehyde/ 2% Sucrose (diluted in PBS) for 10 min at room temperature. Fixed cells were washed three times in PBS to remove any residual amount of fixative and stored at 4 °C before being processed for PLA assay. To block unspecific signals, cells were incubated with 3% BSA in PBS for 30 min at room temperature and then were incubated with the Click-iT reaction cocktail for 30 min at room temperature in order to conjugate the EdU nucleotide with Biotin. The Click-iT cocktail contains 10 µM of Biotin Azide, 10 mM of sodium-ascorbate and 10 mM of copper sulfate (CuSO4) diluted in PBS. For PLA reactions that did not involve detection of EdU positive cells, this step was skipped. After one wash with 1 mL of BSA 3% PBS, glass coverslips were incubated overnight at 4 °C with 30 µL of primary antibodies (Biotin 1:3000, S9.6 1:500, pH2Ax 1:1000, PCNA 1:250, RNA pol II 1:500) raised in either mouse and rabbit, and diluted in PBS supplemented with 1% BSA and 0.1% saponin. After two washes in Buffer A for 5 min each at room temperature, glass coverslips were incubated at 37 °C for 1 hour with 30 µL of Duolink PLA Probes (Minus and Plus) diluted 1:5 in Antibody diluent solution. To tap off any residual amount of probes, slides were washed twice in Buffer A for 5 min each at room temperature and successively incubated with 30 µL of Ligation mix, containing the Ligase enzyme diluted 1:40 in 1x the ligation buffer. After 30 min of incubation at 37 °C, slides were washed twice in Buffer A and then incubated at 37 °C for 100 min with 30 µL of amplification mix containing the Polymerase enzyme diluted 1:80 in the Amplification buffer. Cells were washed twice with Buffer B for 10 min each at room temperature and stained with 0.2 µg/mL of DAPI diluted in PBS for 5 min. Lastly, cells were washed once with Buffer B for 10 min and with 0.01x Buffer B for 1 minute. Glass coverslips were mounted on slides with 5 µL of Mowiol. All the incubation steps were performed in a pre-heated humidity chamber and from the amplification step slides were protected from light. All the washes steps were performed in 12 well plates with 1 mL of washing buffer.

### Image acquisition and processing

Cells were imaged with a Leica Multi-fluorescent wide-field microscope (DM6 B). For each coverslip a sufficient number of cells was recorded by using the oil-immersion 100X objective (NA 1.40). We acquired 15–20 Z-stacks of 0.1 μm thickness for each field of view. The analysis of the images was performed with Fiji software through the implementation of a custom-made macro that allowed to automate the analysis. Briefly, the PLA dots coming from different stacks of the same cell were projected in a 2D space by applying the maximum intensity Z projection method. Cell area was measured by an ImageJ plugin that predicts polygonal shapes around the DAPI signal. PLA dots were counted with the Find Maxima Fiji function.

### Pharmacological studies and IC50 calculation

To perform pharmacological inhibition of kinases involved in DNA damage response, cells were treated with KU55933 (ATM inhibitor, MedChemTronica cat. no. HY-12016), VE821 (ATR inhibitor, MedChemExpress cat. no. HY-14731) or NU7441 (DNA-PK inhibitor, Bio-Techne cat. no. 3712) diluted in DMSO. After 72 h of drug treatment, cell viability was assessed by CellTiter-Glo® Luminescent Cell Viability Assay (Promega G7571). Each measurement was performed in triplicates in 96 well plates. The readout of the luminometer was then used for the IC50 calculation using the Prism software. IC50 was measured by the non-linear fit function of the relative values of luminescence (DMSO-normalized), transformed in logarithmic scale.

### DNA:RNA ImmunoPrecipitation and sequencing (DRIP-seq)

For DRIP-seq experiments, 3×10^6^ cells were seeded in 150 cm plates 24 h being treated with 50 ng/mL of Nocodazole for 18 h. Mitotic cells were retrieved by shaking off the plates, collected at 1000 rounds per minute (rpm) for 5 min at room temperature and washed twice with Phosphate Buffered Saline (PBS). Cells were then reseeded for synchronous cell cycle progression and collected after 18 h from the release for R-loops immunoprecipitation (DRIP-seq). Cell synchronization was assessed by FACS analysis as described for PLA experiments. DRIP was performed according to Sanz and Chedin [43]. Briefly, after trypsinization, 4–7 × 10^6^ late S-phase synchronized cells were resuspended in 1.6 mL of TE buffer supplemented with 50 μL of SDS 20% (wt/vol) and 5 μL of Proteinase K (20 mg/mL) and lysed overnight at 37 °C in a rotating wheel. Lysates were then poured into a phase lock gel tube and mixed with 1.6 mL of Phenol/Chloroform Isoamyl alcohol 25:24:1 for DNA purification. After centrifugation at 1500 g for 5 min, the top aqueous phase containing DNA was mixed with 160 μL of 3 M NaOAc pH 5.2 and 4 mL of absolute Ethanol to precipitate DNA. The insoluble pellet was washed twice with 1.5 mL of 80% Ethanol (10 min each), completely air-dried and then gently resuspended in 125 μL of TE after being incubated on ice for 2 h. The viscous genomic DNA was digested with a cocktail of restriction enzymes (BsrGI, EcoRI, HindIII, SspI, XbaI, 30U each) overnight at 37 °C and fragmented DNA was isolated by pouring it into a phase lock gel light tube, supplemented with 100 μL of water and 250 μL of Phenol/Chloroform Isoamyl alcohol 25:24:1, mixed by gentle inversion and precipitated by centrifugation at 16000 g for 10 min at room temperature. The top aqueous phase containing the DNA was then mixed with 1.5 μL of glycogen, 25 μL of 3 M NaOAc pH 5.2 and 625 μL of Ethanol and incubated for at least one hour at −20 °C to increase precipitation yields. DNA was then spun down at 16000 g for 35 min at 4 °C, washed with 200 μL of room temperature 80% Ethanol, air-dried, resuspended in 50 μL of TE buffer and incubated on ice for 1 hour. 6 μg of digested DNA were mixed with 2 μg of mouse DNA (from S-late phase synchronized NIH-3T3 cells, used as spike-in for internal normalization for the sequencing) in a final volume of 500 μL of TE buffer to proceed with R-loops immunoprecipitation. As control for the specificity of the signal, part of the extracted DNA was pre-treated with 4 μL of RNase H (NEB) for 4.5 h at 37 °C to eliminate R-loops. For RNA-DNA hybrids isolation, 450 μL of the DNA (pre-treated with RNAseH and not) was mixed with 52 μL of 10X binding buffer and 20 μL of S9.6 antibody (Kerafast, cat. No. ENH001) and incubated for 16 h at 4 °C while gently inverting on a mini-tube rotator. DNA was then incubated with 100 μL of agarose beads (pre-washed twice with 700 μL of 1X binding buffer) for 2 h at 4 °C while gently inverting on a mini-tube rotator. Once spun-down at room temperature at 1100 g to discard the supernatant, beads were washed twice with 750 μL of 1X binding buffer, centrifuged for one minute at 1100 g and resuspended in 300 μL of DRIP elution buffer supplemented with 7 μL of proteinase K (20 mg/mL). After 45 min of incubation at 55 °C in a rotating wheel, beads were centrifuged for one minute at 1100 g and the supernatant collected for DNA isolation with Phenol/Chloroform Isoamyl alcohol. DNA pellets were air dried for 15 min, resuspended in 50 μL of RNase-free TE buffer and kept on ice for 15 to 30 min. DNA was sonicated with Pico Bioruptor with 6 cycles of 15 sec ON / 90 sec OFF at high intensity in 4 °C water bath to get fragments of 300 bp. After quantification with Qubit, 10 ng of DNA were used for library preparation using the New England NEBNext Ultra II kit (New England Biolabs, product No: E7645S) and AMPure XP beads (Beckman Coulter, product No: A63882). Libraries were sequenced on a Novaseq 6000 sequencer (50 bp, paired-ends, 30 millions reads).

### 5-ethynyluridine labeling of nascent transcripts and sequencing (EU-seq)

Cells were synchronized as described for DRIP-seq experiment with the exception of being pulsed with 0.5 mM of EU (5-ethynyl-uridine, Jena Biosciences, cat. no. CLK-N002-10) for 2 h to label the newly synthesized RNA before harvest. After RNA extraction (*Quick-*RNA^TM^ Miniprep Kit, Zymo Research, cat. #R1055), 45 μg of U2OS RNA were mixed with 5 μg of NIH-3T3 RNA (from asyncronous EU-pulsed cells) used as spike-in for post-sequencing normalization. EU-labelled RNA was isolated by Click-it reaction chemistry (Invitrogen Click-iT® Nascent RNA Capture Kit). Briefly, the RNA was mixed 1:1 with the Click-it solution (171 mM Tris HCl pH8, 8 mM CuSO4, 200 mM Sodium-L-Asc, 0.1 mM Biotin Azide) and incubated 30 min at room temperature. Then, 751 μL of precipitation mix (1 μL of Glycogen, 50 μL of Ammonium acetate 7.5 M, 700 μL of cold 100% ethanol) were added to the RNA overnight at −80 °C. The RNA was isolated by centrifugation at 13,000 x g for 20 min at 4 °C. After 2 washes with 700 μL of 75% ethanol, the pellet was air-dried for 10 min and then resuspended in 100 μL of Ultra‑Pure™ DNase/RNase-free distilled water supplemented with RNaseOUT inhibitor (diluted 1:100, Invitrogen, cat. no. 10777-019). To isolate the EU-labelled RNA, 50 μL of Dynabeads MyOne streptavidin C1 (Invitrogen, cat. no. 65001) for each RNA sample were washed twice with Binding and Washing Buffer 1X (5 mM Tris-HCl pH 7.5, 0.5 mM EDTA, 1 M NaCL, 0.5% Tween-20), twice with Solution A (0.1 M NaOH, 0.05 M NaCl) and twice with Solution B (0.1 M NaCl). Then, beads were resuspended into twice the original volume with Binding and Washing Buffer 2X and mixed with an equal volume of EU-labelled RNA, previously heated at 70 °C for 5 min to remove secondary structures. The mix was incubated for 30 min on a rotating wheel at room temperature and placed on the magnet for 2 min to remove the flow through. After 3 washes with Binding and Washing Buffer 1X and one wash with water, the EU-labelled RNA was eluted with 55 µL of 2% β-mercaptoethanol (Calbiochem, cat. no. 444203) diluted in 10 mM Tris-HCl pH 8 and incubated for 1 hour at room temperature. The supernatant was dried in a Speed-Vac machine for 25 min, cooled-down on ice for 10 min and dried again for 15 min. After quantification by Bioanalyzer, 200 ng of EU-RNA were used for library construction by TruSeq Stranded Total RNA by Illumina (Illumina, cat. no. 20020598). Libraries were sequenced on a Novaseq 6000 sequencer (50 bp, paired-ends, 35 millions reads).

### Breaks Labeling in situ and sequencing (sBLISS)

Cells were synchronized as described for DRIP-seq experiment. sBLISS experiments were performed according to Bouwman et al. [56]. Briefly, late S-phase synchronized cells (harvested 18 h after the release) were diluted to 10^6^ cells per mL, filtered with a 70 µm cell strainer and fixed in PFA 2% for 10 min at room temperature while gently rotating the tube on a roller–shaker. The fixative solution was quenched with 125 mM glycine for 5 min at room temperature and then for 5 min on ice. Cells were collected by centrifugation at 100–300 x g for 10 min at 4 °C, washed twice with 10 mL of ice-cold PBS and stored at 4 °C at the final concentration of 1.5 ×10^6^ cells/mL. To prepare the sBLISS template, cells were washed with ice-cold PBS, collected at 300 g for 5 min at 4 °C, incubated with 300 μL of LB1 for 60 min on ice, collected at 300 g for 5 min at 4 °C and resuspended in 300 μL of pre-warmed LB2 for 60 min at 37 °C while gently shaking at 400 rpm. After two washes with 300 μL of pre-warmed RT CSTX buffer, DSBs were blunted by incubating samples with 200 µL of blunting mix (150 µL of water, 20 µL of Blunting Buffer 10X, 2 μL of 10 mg/mL BSA, 20 µL of 1 mM dNTPs mix, 8 µL Blunting enzyme) for 60 min at room temperature with 400 rpm shaking. Then, cells were washed twice with 400 μL of CSTX buffer, centrifuged at 300 g for 5 min at room temperature and mixed first with 4 μL of a unique double stranded sBLISS adaptor (freshly annealed), then with 96 μL of the ligation mix (66 μL of water, 10 μL of T4 DNA ligase Buffer 10X, 12 μL of 10 mM ATP, 3 μL of 550 mg/mL BSA, 5 μL of T4 DNA ligase). After an overnight incubation at 16 °C, cells were washed twice with 400 μL of CSTX buffer at room temperature, collected by centrifugation at 300 x g for 10 min at room temperature and incubated overnight at 55 °C in shaking (800 rpm) with 150 μL of Tail buffer supplemented with 10 μL of Proteinase K. The next day, 10 μL of fresh Proteinase K were added for an extra hour of protein digestion at 55 °C, then samples were incubated at 95 °C for 10 min to inactivate the enzyme. Once the samples were cooled at room temperature, DNA was isolated through Phenol/Chloroform and precipitated through ice-cold absolute Ethanol. The isolated BLISS template was resuspended in 102 μL of TE at 50 °C in 1100 rpm for 15 min and fragmented with Covaris sonicator at 4 °C for 55 seconds (peak power 140, cycle/burst 200, duty factor 10). To purify BLISS template, 100 μL of fragmented DNA were incubated with 80 μL of AMPure XP beads for 10 min at room temperature, DNA-beads complex were isolated through the DynaMag magnetic, washed twice with 180 μL of freshly prepared Ethanol 80% and after being air-dried beads were resuspended in water to elute the DNA. DNA concentration and size were evaluated by Qubit quantification (DNA High Sensitive) and Tape station on 1:10 diluted DNA. 300 ng of DNA were diluted in a final volume of 7.5 μL of water, mixed with 8 μL of rNTP, 2 μL of Reaction Buffer 10X, 2 μL of T7 Enzyme, 0.5 μL RiboSafe Rnase Inhibitor and incubated for 14 h at 37 °C in a thermal cycler with the lid set to 70 °C for the in-vitro transcription (IVT). 1 μL of DNAse I (2U/μL) was added to each IVT reaction and incubated for 15 min at 37 °C to degrade the DNA template. Each sample was diluted with 9 μL of water (to reach a final volume of 30 μL) and then mixed with 30 μL of RNACleanXP beads to isolate RNA through a magnetic stand. RNA was eluted from beads in 8 μL of water and run on a TapeStation to evaluate the concentration and the size of the library. 6 μL of purified RNA was incubated first with 1 μL of RA3 adaptor (10 μM) for 2 min at 70 °C to solve any secondary structure, then on ice for at least 1 minute and finally mixed with 1 μL of T4 RNA Ligase Reaction Buffer 10X, 1 μL of RNAseOUT Recombinant Ribonuclease Inhibitor and 1 μLT4 RNA Ligase 2 truncated. After adaptors ligation was carried out for 2 h at 25 °C RNA undergoes to reverse transcription: RNA was incubated with 2 μL of RTP (10 μM) for 2 min at 70 °C to solve any secondary structure, and after a short incubation on ice samples were mixed with 13 μL of Reverse Transcription master mix (1 μL of water, 5 μL of RT Buffer 5X, 1 μL of 25 mM dNTPs, 2 μL of 100 mM DTT, 2 μL of RNaseOUT Recombinant Ribonuclease Inhibitor, 2 μL of SuperScript IV Reverse Transcriptase) and incubated in a thermal cycler with the lid set to 70 °C with the following program: 50 min at 50 °C, 10 min at 80 °C, 4 °C hold. The library was indexed by using 20 μL of 10 μM RPIX library-specific indexed primers and mixed with 135 μL of water, 20 μL of 10 μM RP1 COMMON primer, 200 μL of NEBNext ultra II Q5 Master Mix for 8 cycles of PCR amplification (10 seconds of denaturation at 98 °C, 30 seconds of annealing at 60 °C, 45 seconds of extension 65 °C plus a final cycle of 10 min extension at the same temperature). The amplified libraries were then diluted in a final volume of 400 μL, purified with 180 μL of pre-warmed AMPure XP Beads to discard products larger than 600-700 bp, then with 120 μL of AMPure XP Beads, eluted in 51 μL of water and purified with 40 uL of AMPure XP Beads. DNA was finally eluted in 20 μL of water and checked by Qubit quantification (DNA High Sensitive) and TapeStation. Libraries were sequenced on a Novaseq 6000 sequencer (50 bp, paired-ends, 100 millions reads).

### Bioinformatic analyses

#### BLISS analysis and identification of BLISS positive regions

Raw sequencing data were demultiplexed on Illumina’s BaseSpace to generate FASTQ files, which were processed using the sBLISS pipeline previously described in detail [56]. This pipeline first identifies reads containing the expected sBLISS prefix consisting of an 8 nt UMI followed by an 8 nt sample barcode, using SAMtools (v1.10) [57] and Scan for Matches [58], allowing at most one mismatch in the barcode. Prefixes are clipped off and stored, while the trimmed reads are aligned to the human reference genome (GRCh38/hg38) using BWA-MEM22 (v0.7.17-r1188) with default options [59]. Reads with mapping quality score lower than 30 and PCR duplicates are removed by searching for adjacently mapped reads (at most 10 bp apart along the reference genome) with at most one mismatch in their UMI sequence. The output of the pipeline is a list of unique DSB locations and corresponding number of unique UMIs identified at each position. Given the wide range in terms of number of DSB locations between samples, we decided to perform down sampling to match the sample with the least elements.

We used a custom R script, previously designed [14] to identify genomic regions enriched for BLISS signals (BLISS+ regions). Briefly, the hg38 genome was divided in 2 kb bins. For each bin, we calculated the coverage (number of BLISS UMI for each bin) using the GRbaseCoverage2 and the makeMatrixFrombaseCoverage functions from the ChroKit framework [60]. Then, based on the distribution of the BLISS signal coverage across bins, we set an arbitrary threshold on the BLISS signal (threshold of the bin coverage=22) above which bins were called as BLISS + . BLISS+ bins were then collapsed into BLISS+ positive regions by merging BLISS+ bins that were separated by a maximum gap of 2 kb. Samples for the Myc-activated condition in U2OS-MycER-shNT were also available from a former publication [14] with the accession codes GSM7549772 and GSM75497723 (series GSE236553) in the GEO database. For those samples, genomic coordinates were converted from hg19 to hg38 assembly using CrossMap [61], both for bed and bigWig files. For genomic analyses we used the union of the BLISS+ regions identified in the replicates for each condition (OHT, shSETX, OHT+shSETX).

Heatmaps and figures were generated using the Chrokit framework [60].

#### EdU-seq-HU analysis

Published data [14] are available in the GEO database under accession code GSE236553 were used. Genomic coordinates for processed files (bed and bigWig), for U2OS-MycER-shNT with and without Myc-induction, were converted from hg19 to hg38 assembly using CrossMap [61].

#### EU-seq analysis

Paired-end reads were aligned using STAR with the following command: STAR --runThreadN 8 --runMode alignReads --outSAMtype BAM SortedByCoordinate --genomeDir <index > --readFilesIn reads_R1.fq reads_R2.fq. Samples were first aligned to hg38 (signal) and then to mm10 (spike-in). Reads that aligned to both genome assemblies were excluded from the following steps. The number of counts for each gene were computed using featureCounts -T 2 -p -P -a gtffile. Differentially expressed genes between experimental conditions (OHT, shSETX, OHT+shSETX) and not treated samples were calculated using DESeq2 R Bioconductor package where the size factors were calculated using the spike-in counts. All the genes with an adjusted *p*-value < 0.05 were defined as DEG up or DEG down, respectively. Genes were considered expressed if they had a value for *baseMean* > 32. BigWig files were generated using the bamCoverage function of deeptools v. 3.5.1 with the spike-in reads used as scaleFacor. A bigWig file with the average coverage for each condition was also produced using WiggleTools [62].

#### DRIP-seq analysis

Paired-end reads were aligned using the BWA v.0.6.2 tool [59] with default settings, using hg38 genome assembly. Samples were first aligned to hg38 (signal) and then to mm10 (spike-in). Reads that aligned to both genome assemblies were excluded from the following steps. The number of counts for each gene were computed using featureCounts -T 2 -p -P -a gtffile, where the gtffile was modified enlarging each gene coordinates by 10% of the gene length before its TSS and after its TES in order to better capture the DRIP-seq enrichment which can be found bordering the transcripts. DESeq2 R Bioconductor package was used to find genes with different DRIP-seq enrichment between experimental conditions (OHT, shSETX, OHT+shSETX) and not treated samples. Each comparison used spike-in counts to compute DESeq2 size factors. All the genes that resulted to have an adjusted *p*-value < 0.05 were defined as DRIP-DEG up or DRIP-DEG down, respectively. As a reference control group for genes with no change in DRIP-seq enrichment, a random sample of 500 genes with adjusted *p*-value > 0.5 was selected.

BigWig files were generated using the bamCoverage function of deeptools v. 3.5.1 with the spike-in reads used as scaleFacor. A bigWig file with the average coverage for each condition was also produced using WiggleTools [62].

## Supplementary information

supplemental figures

materials and reagents

RNA-seq data

uncropped western blots

## Data Availability

Genomic data are deposited in GEO (GEO accession number: GSE275922 [BLISS], GSE275923 [DRIP-seq], GSE275924 [EU-seq]). Scripts and codes are available upon request.
