# Correction: Senataxin prevents replicative stress induced by the Myc oncogene

**DOI:** 10.1038/s41419-025-07671-4

**Published:** 2025-05-12

**Authors:** Silvia Sberna, Marco Filipuzzi, Nicola Bianchi, Ottavio Croci, Federica Fardella, Chiara Soriani, Sara Rohban, Sara Carnevali, Alessandra Alberta Albertini, Nicola Crosetto, Simona Rodighiero, Arianna Chiesa, Laura Curti, Stefano Campaner

**Affiliations:** 1 Center for Genomic Science of IIT, CGS@SEMM (Istituto Italiano di Tecnologia at European School of Molecular Medicine), Fondazione Istituto Italiano di Tecnologia (IIT), 20139 Milan, Italy; 2 Imaging Unit, Department of Experimental Oncology, IEO, European Institute of Oncology IRCCS, Milan, Italy; 3 Human Technopole, Viale Rita Levi-Montalcini 1, 20157 Milan, Italy; 4 Department of Microbiology, Tumor and Cell Biology, Karolinska Institutet, Stockholm, SE 17165 Sweden; 5 Science for Life Laboratory, Tomtebodavägen 23A, Solna, SE 17165 Sweden; 6 Department of Molecular Medicine, University of Padua, Padua, Italy

**Keywords:** Checkpoints, Oncogenes

Correction to: *Cell Death and Disease* 10.1038/s41419-025-07485-4, published online 19 March 2025

In this article the affiliation of Chiara Soriani & Simona Rodighiero “^2^Imaging Unit, Department of Experimental Oncology, European Institute of Oncology (IEO), Milan, Italy” needs to be updated to “Imaging Unit, Department of Experimental Oncology, IEO, European Institute of Oncology IRCCS, Milan, Italy”.

The original article has been updated.

