# Supplementary material for: Senataxin prevents replicative stress induced by the Myc oncogene
Source: Cell Death Dis. 2025 Mar 19;16(1):187. doi: 10.1038/s41419-025-07485-4 (PMC11923212; doi:10.1038/s41419-025-07485-4)
Supplement: Supplementary file 1 — supplemental figures [file 41419_2025_7485_MOESM1_ESM.docx]

**Senataxin prevents replicative stress induced by the Myc oncogene**

Silvia Sberna^1^, Marco Filipuzzi^1^, Nicola Bianchi^1^, Ottavio Croci^1^, Federica Fardella^1^, Chiara Soriani^2^, Sara Rohban^1^, Sara Carnevali^1^, Alessandra Alberta Albertini^3^, Nicola Crosetto^3,4,5^, Simona Rodighiero^2^, Arianna Chiesa^1^, Laura Curti^1^ and Stefano Campaner^1*^

^1^Center for Genomic Science of IIT, CGS@SEMM (Istituto Italiano di Tecnologia at European School of Molecular Medicine), Fondazione Istituto Italiano di Tecnologia (IIT), 20139 Milan, Italy.

^2^Imaging Unit, Department of Experimental Oncology, European Institute of Oncology (IEO), Milan, Italy.

^3^ Human Technopole, Viale Rita Levi-Montalcini 1, 20157 Milan, Italy

^4^Department of Microbiology, Tumor and Cell Biology, Karolinska Institutet, Stockholm, SE-17165, Sweden.

^5^Science for Life Laboratory, Tomtebodavägen 23A, Solna, SE-17165, Sweden.


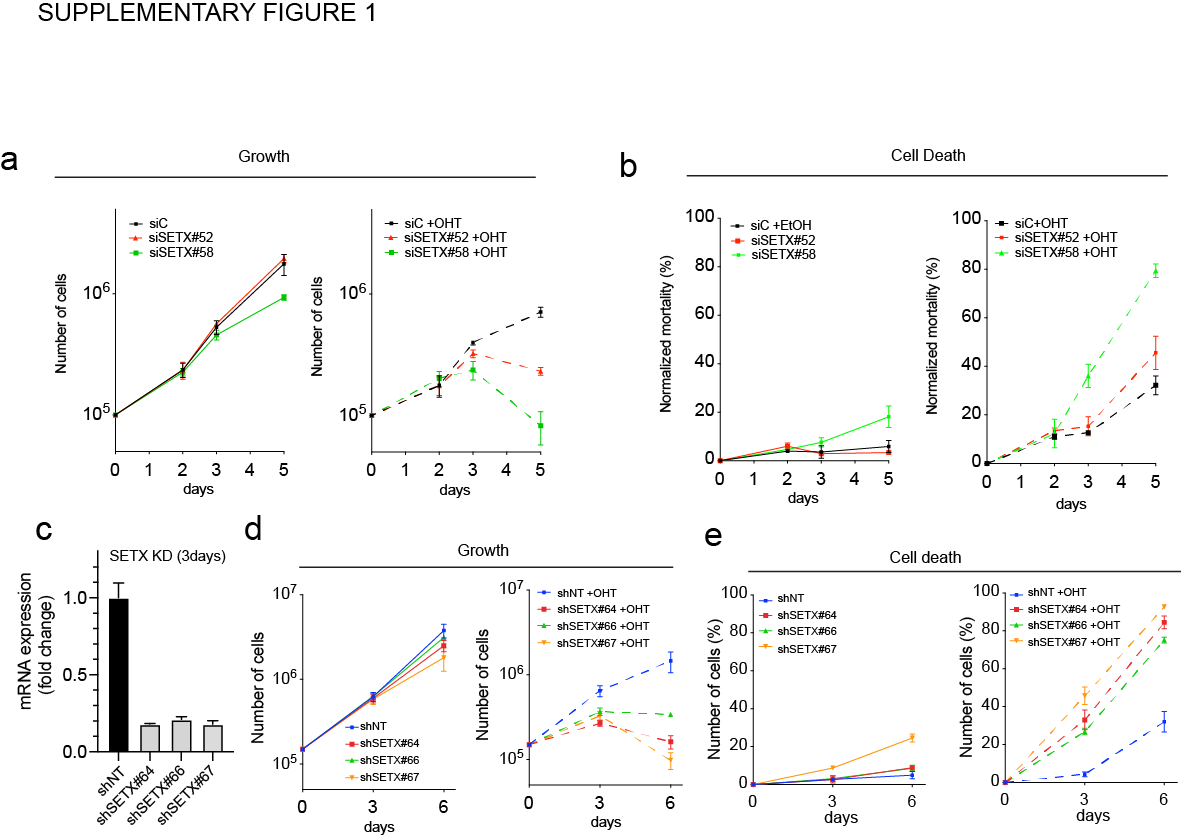


**Supplementary figure 1.** **Loss of SETX is synthetic lethal with MycER activation.** (a, b) Survival and cell death curves of U2OS-MycER cells transfected with the indicated siRNAs. Data points are the average of three replicates (n=3) and error bars are the Stdv. (c) Bar graph of the mRNA levels determined by RT-qPCR. n=3, data points: average, error-bars: stdv. (d, e) Survival and cell death curves of U2OS-MycER cells infected with the indicated shRNAs. Data points are the average of three replicates (n=3) and error bars are the Stdv.


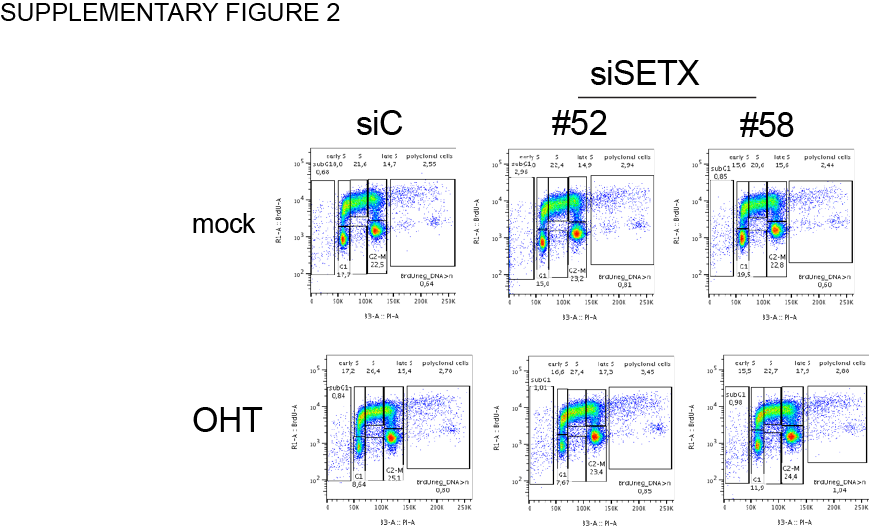

**Supplementary figure 2. Analysis of cell cycle distribution and cell cycle progression.**

Upper panel, bi-parametric FACS analysis of U2OS-MycER cells pulse-labeled with BrdU 24 hours after transfection with the indicated siRNAs. OHT was used to activate MycER. Lower panel, bi-parametric FACS analysis of U2OS-MycER cells released from mitotic arrest and pulse-labeled with BrdU before collection.


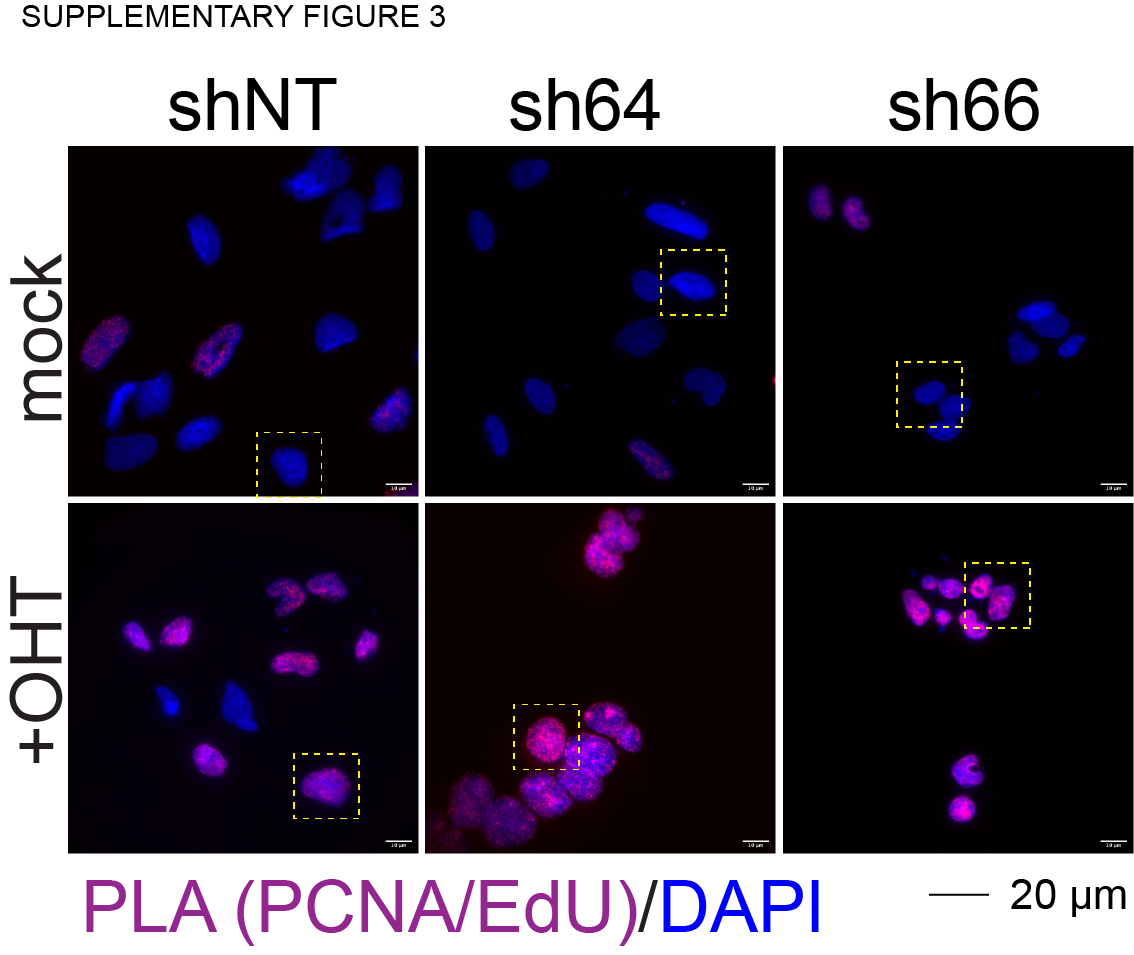


**Supplementary figure 3. Replication foci analysis by assessing the proximity between nascent DNA and the replisome (PCNA) by PLA.**

Representative micrographs of PLA-foci in U2OS-MycER cells carrying shRNAs against SETX (#64, #66) or a non-targeting shRNA (shNT). In blue, the nuclei stained with DAPI; in red, the PLA signal. Highlighted cells in each panel are shown in figure 4. Cells were seeded in the presence of doxycycline to induce the expression of the shRNA and of OHT to activate MycER. Ethanol was used as mock treatment. After 48 hours, cells were synchronized at prometaphase by 100 ng/mL of Nocodazole for 8 hours. Synchronized cells were released and collected after 18 hours (late S phase). Before fixation, cells were pulsed with 25 µM of EdU to label the newly synthesized DNA.

**
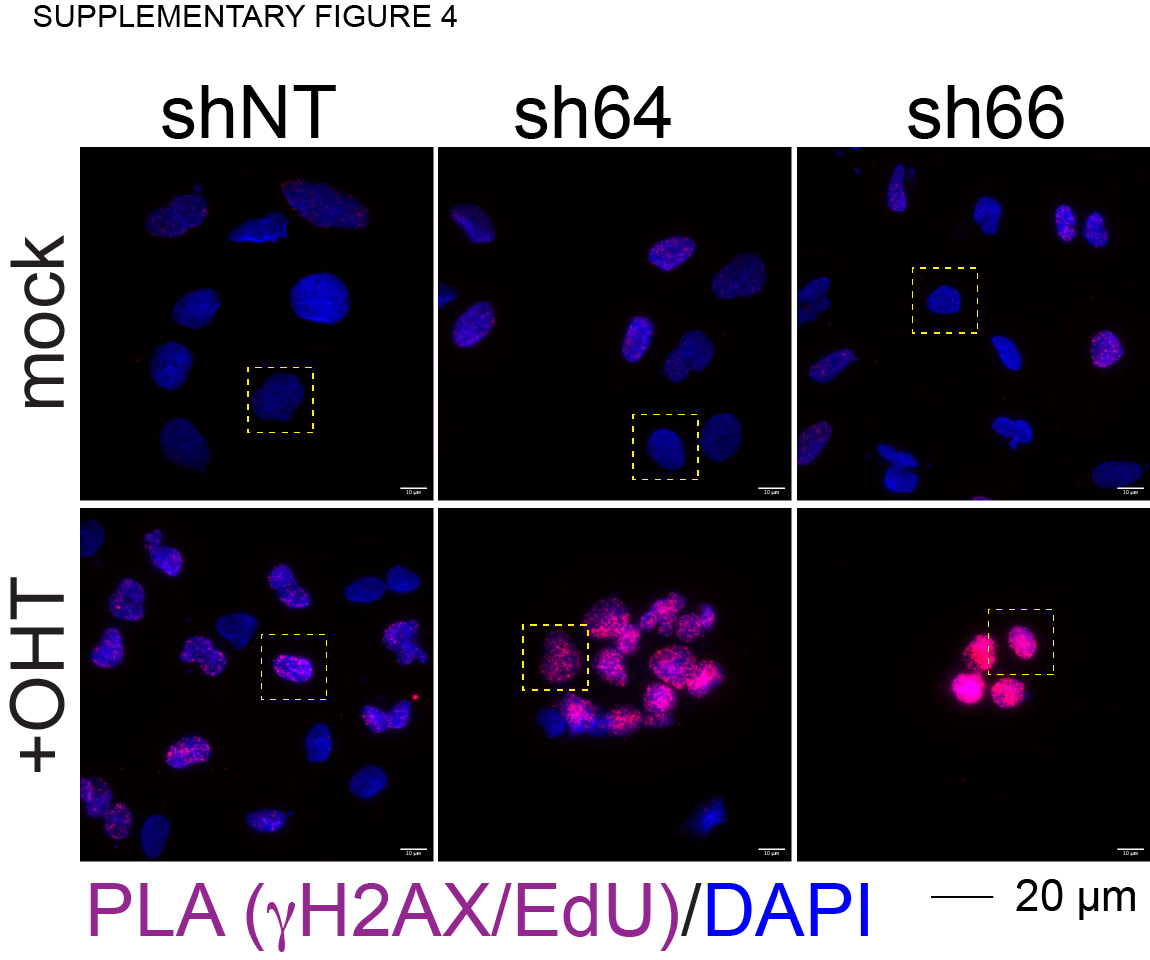
**

**Supplementary figure 4. Analysis of proximity of DDR foci (γH2Ax) and newly synthesized DNA (EdU-labeled DNA) by PLA.**

Representative micrographs of PLA-foci in U2OS-MycER cells carrying shRNAs against SETX (#64, #66) or a non-targeting shRNA (shNT). In blue, the nuclei stained with DAPI; in red, the PLA signal. Highlighted cells in each panel are shown in figure 4. Cells were seeded in the presence of doxycycline to induce the expression of the shRNA and of OHT to activate MycER. Ethanol was used as mock treatment. After 48 hours, cells were synchronized at prometaphase by 100 ng/mL of Nocodazole for 8 hours. Synchronized cells were released and collected after 18 hours (late S phase). Before fixation, cells were pulsed with 25 µM of EdU to label the newly synthesized DNA.

**
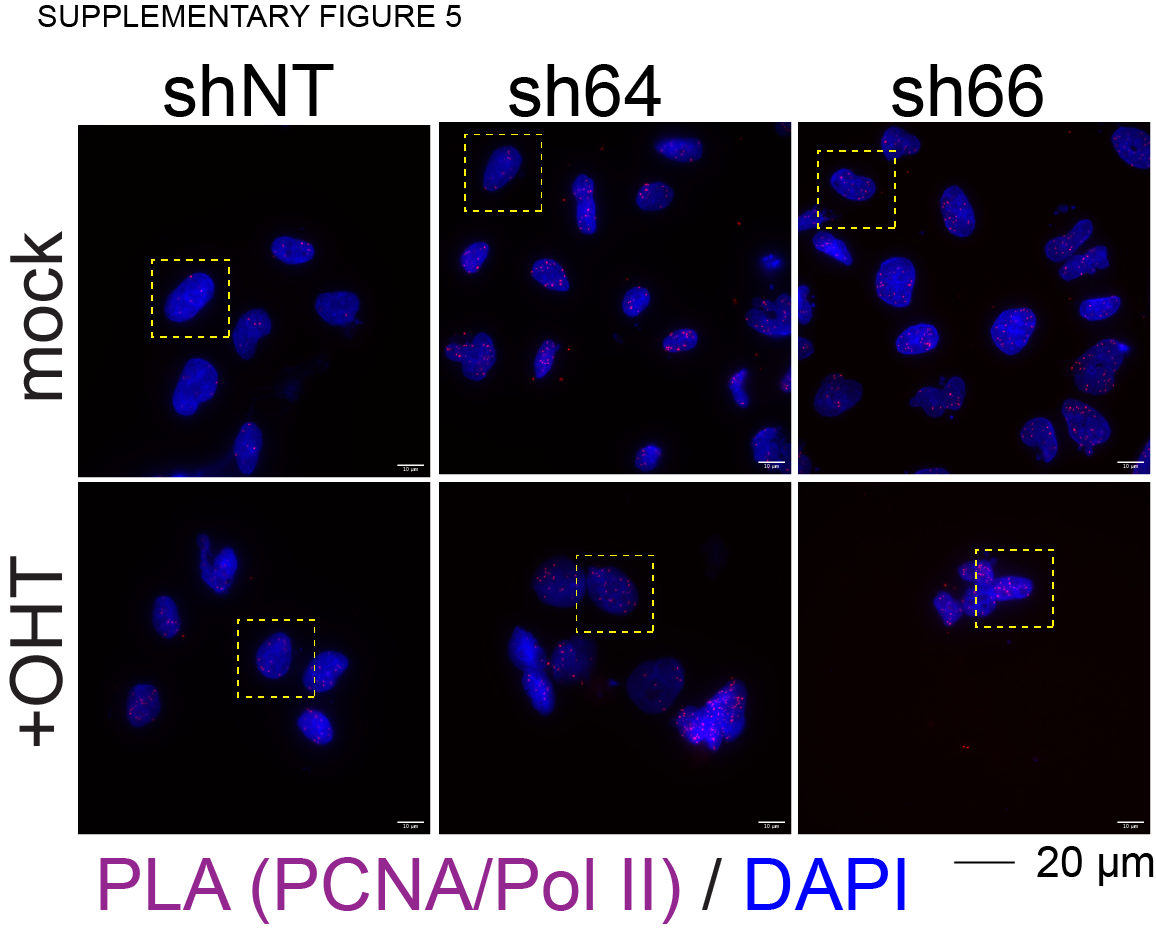
**

**Supplementary figure 5. Assessment of TRCs by PLA**

TR-conflicts were measured by PLA assay to assess the proximity between RNA pol II and PCNA protein. Representative micrographs of PLA-foci in U2OS-MycER cells carrying shRNAs against SETX (#64, #66) or a non-targeting shRNA (shNT). In blue, the nuclei stained with DAPI; in red, the PLA signal. Highlighted cells in each panel are shown in figure 5. Cells were seeded in the presence of doxycycline to induce the expression of the shRNA and of OHT to activate MycER. Ethanol was used as mock treatment. After 48 hours, cells were synchronized at prometaphase by 100 ng/mL of Nocodazole for 8 hours. Synchronized cells were released and collected after 18 hours (late S phase).


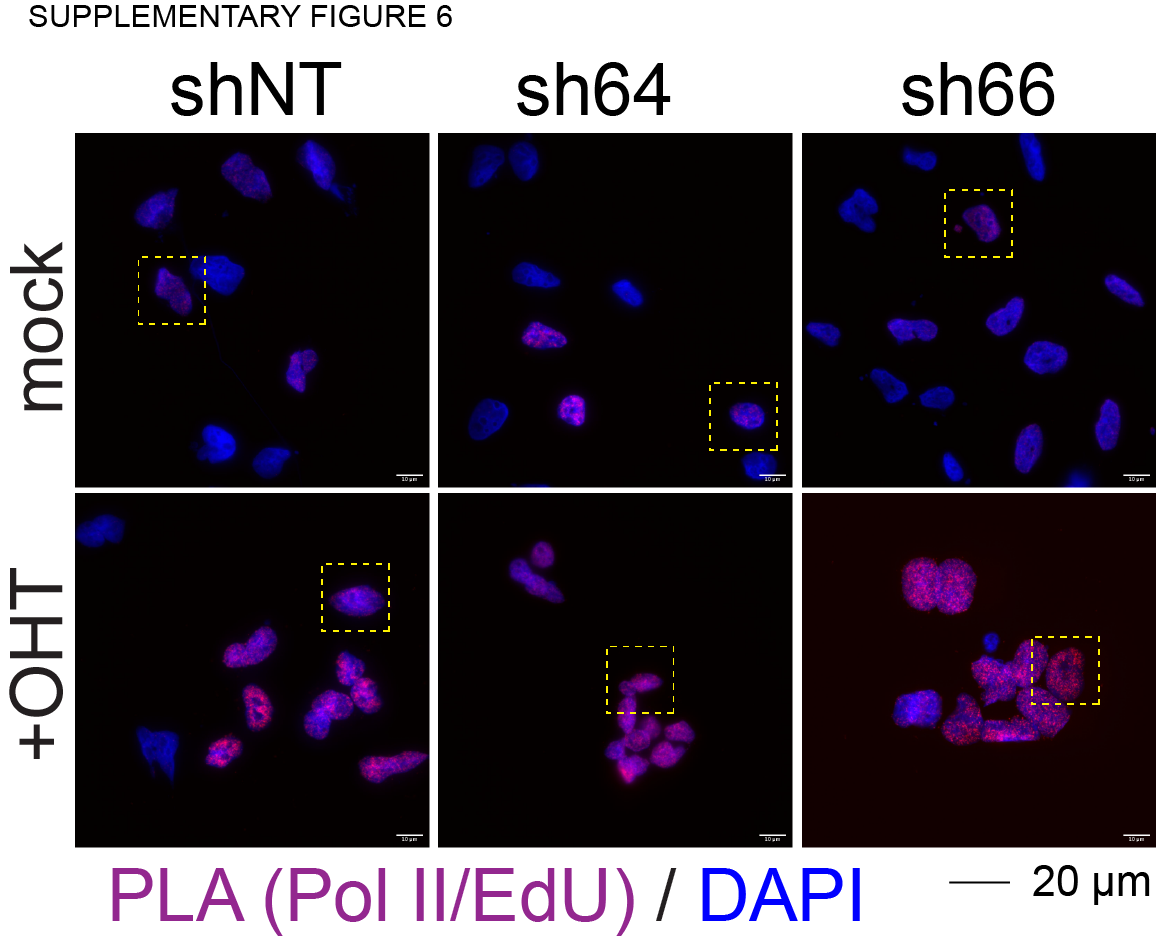


**Supplementary figure 6. Assessment of TRCs by PLA on pulse labelled nascent DNA**

TR-conflicts were measured by PLA assay to assess the proximity between RNA pol II and and newly synthesized DNA (EdU labeled). Representative micrographs of PLA-foci in U2OS-MycER cells carrying shRNAs against SETX (#64, #66) or a non-targeting shRNA (shNT). In blue, the nuclei stained with DAPI; in red, the PLA signal. Highlighted cells in each panel are shown in figure 5. Cells were seeded in the presence of doxycycline to induce the expression of the shRNA and of OHT to activate MycER. Ethanol was used as mock treatment. After 48 hours, cells were synchronized at prometaphase by 100 ng/mL of Nocodazole for 8 hours. Synchronized cells were released and collected after 18 hours (late S phase). Before fixation, cells were pulsed with 25 µM of EdU to label the newly synthesized DNA.

**
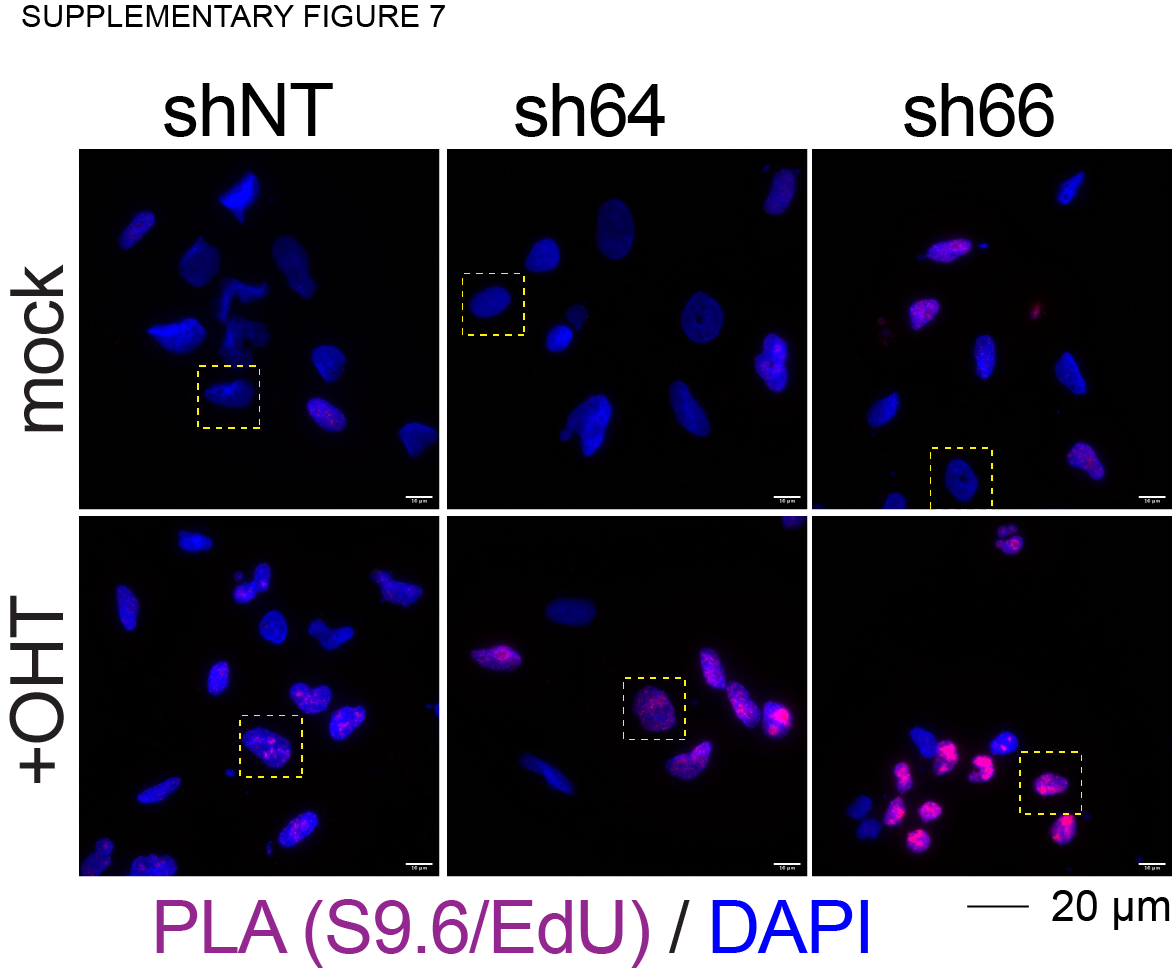
**

**Supplementary figure 7. Proximity of RNA-DNA hybrids to nascent DNA.**

Representative micrographs of PLA-foci in U2OS-MycER cells carrying shRNAs against SETX (#64, #66) or a non-targeting shRNA (shNT). In blue, the nuclei stained with DAPI; in red, the PLA signal. Highlighted cells in each panel are shown in figure 6. Cells were seeded in the presence of doxycycline to induce the expression of the shRNA and of OHT to activate MycER. Ethanol was used as mock treatment. After 48 hours, cells were synchronized at prometaphase by 100 ng/mL of Nocodazole for 8 hours. Synchronized cells were released and collected after 18 hours (late S phase). Before fixation, cells were pulsed with 25 µM of EdU to label the newly synthesized DNA. Fixed cells were stained for PLA to measure the proximity of RNA-DNA hybrids (S9.6) to nascent DNA (EdU labeled DNA).


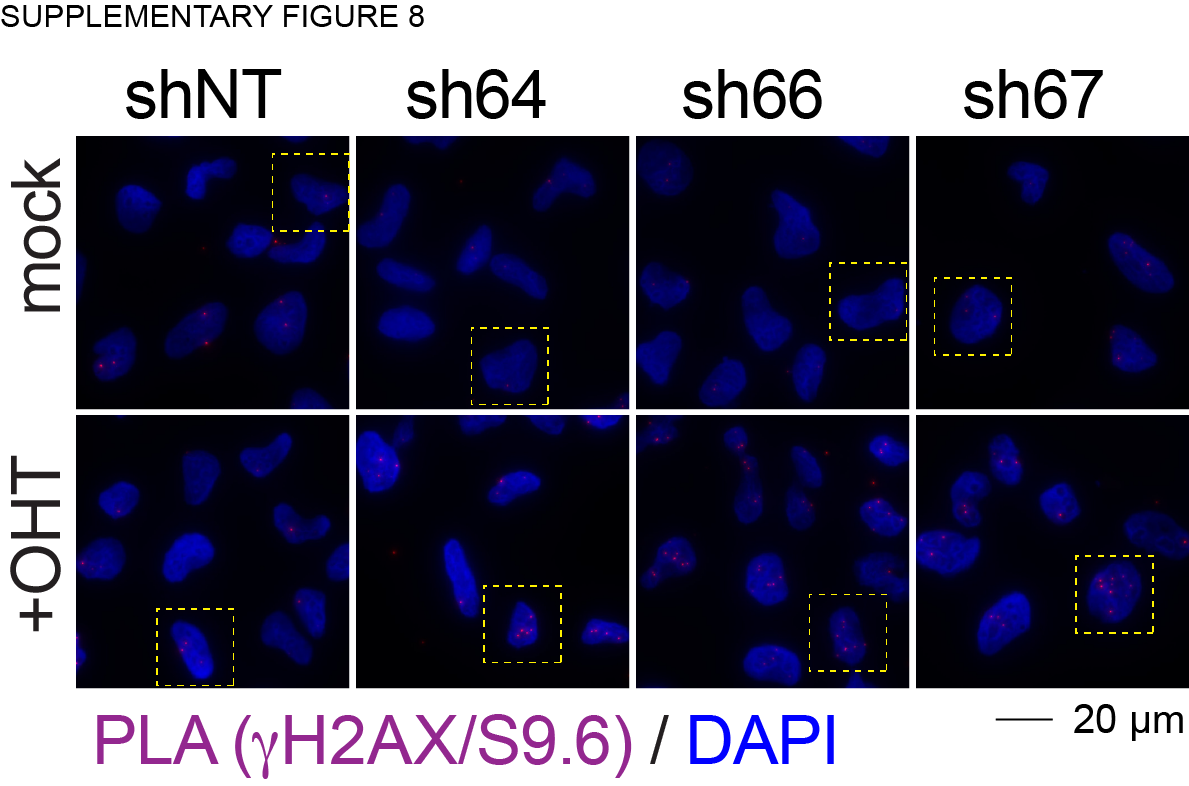


**Supplementary figure 8. Proximity of RNA-DNA hybrids and DDR foci.**

Representative micrographs of PLA-foci in U2OS-MycER cells carrying shRNAs against SETX (#64, #66 and #67) or a non-targeting shRNA (shNT). In blue, the nuclei stained with DAPI; in red, the PLA signal. Highlighted cells in each panel are shown in figure 6. Cells were seeded in the presence of doxycycline to induce the expression of the shRNA and of OHT to activate MycER. Ethanol was used as mock treatment. After 48 hours, cells were synchronized at prometaphase by 100 ng/mL of Nocodazole for 8 hours. Synchronized cells were released and collected after 18 hours (late S phase). Fixed cells were stained for PLA to measure the proximity of RNA-DNA hybrids (S9.6) to DDR foci (ɣH2Ax).


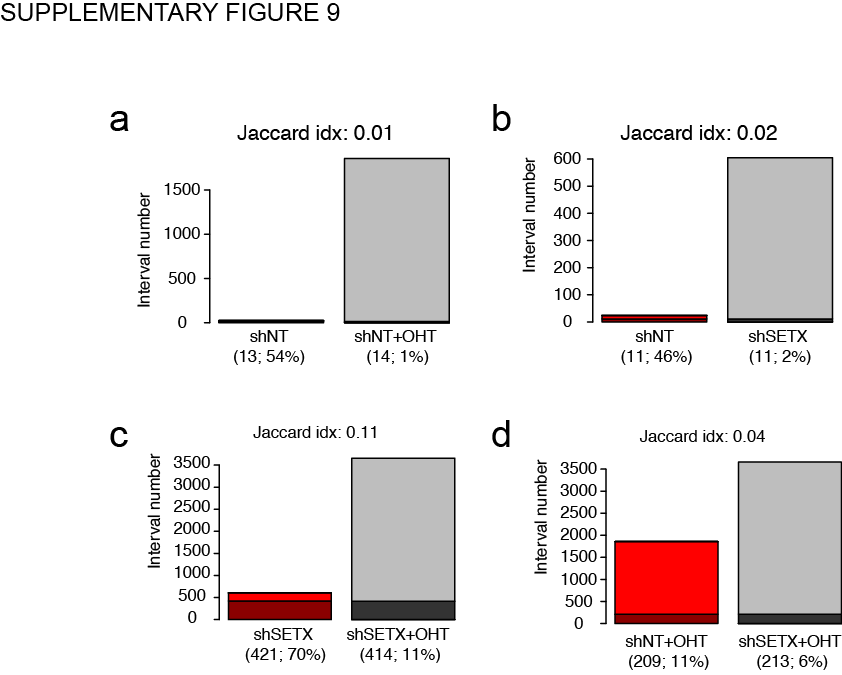


**Supplementary figure 9.** (a-d) Bar graph of the overlap of BLISS+ regions. The number and fraction of the overlapping regions is reported below each histogram.


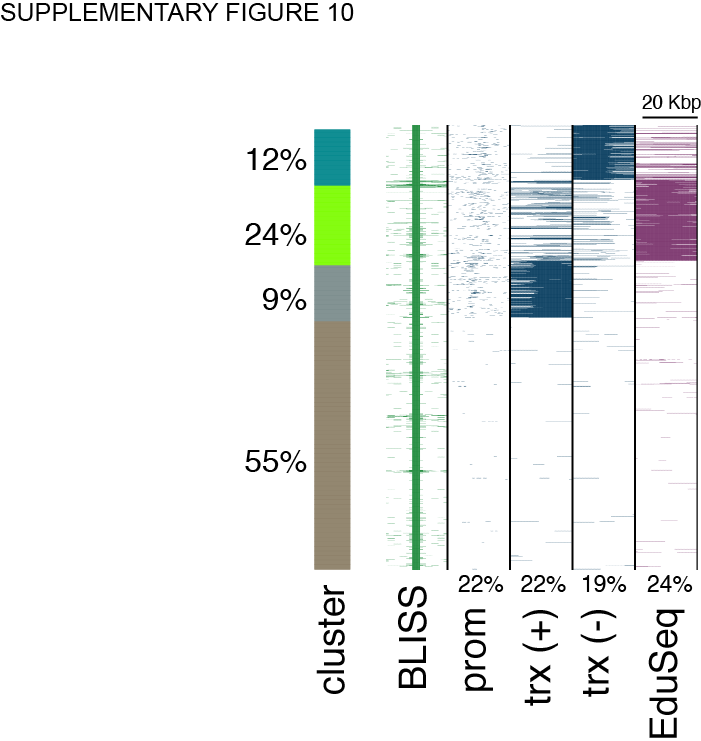


**Supplementary figure 10. Analysis of DSBs detected upon MycER activation.**

Heatmap of the BLISS+ regions detected upon MycER activation (shNT, +OHT), centered on the BLISS+ regions and clustered by transcripts and ERRs (detected by EdU-HU-seq).

**
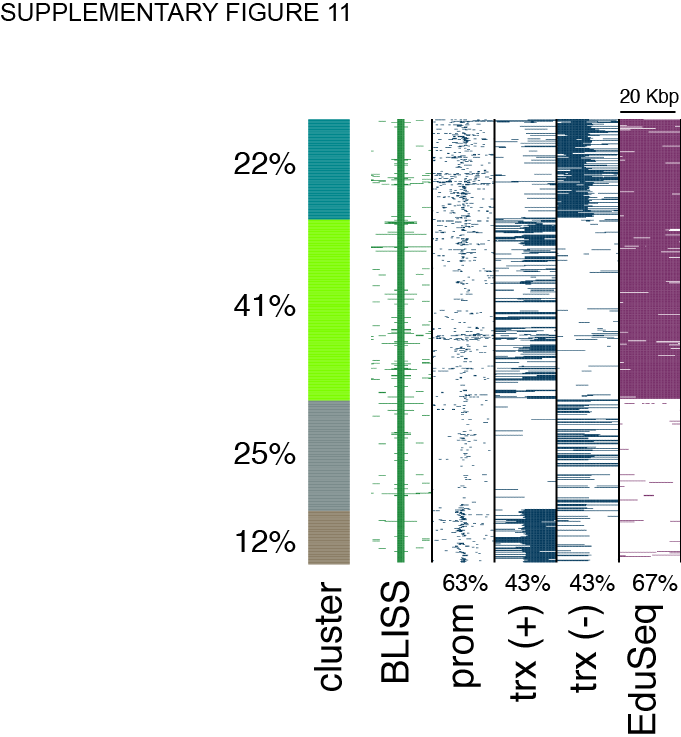
**

**Supplementary figure 11. Analysis of DSBs detected upon SETX silencing.**

Heatmap of the BLISS+ regions detected upon MycER activation (ShNT, +OHT), centered on the BLISS+ regions and clustered by transcripts and ERRs (detected by EdU-HU-seq).


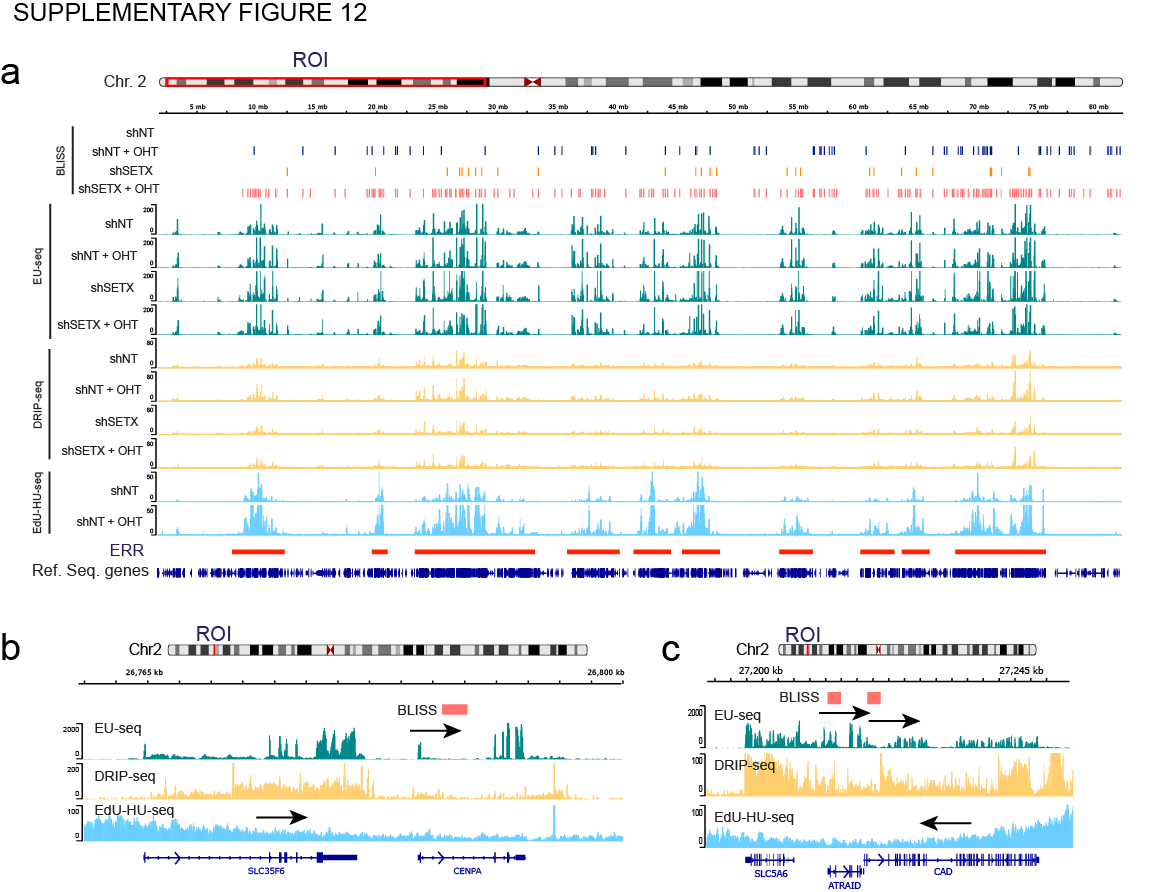


**Supplementary figure 12. Genome browser snapshots.** Transcribed loci were detected by EU-seq, RNA-DNA hybrids by DRIP-seq and early replicated regions (ERRs) by EdU-HU-seq.

(a) Chromosome level view of the distribution of BLISS+ regions along the short arm of chromosome 2. ROI: the displayed region of interest.

(b,c) Gene level view showing that BLISS+ regions (DSBs) are nested between ERRs and S-phase transcribed genes. Arrows indicate the direction of RNA synthesis (based on gene annotation) and DNA synthesis (based on the EdU-HU signal).

**
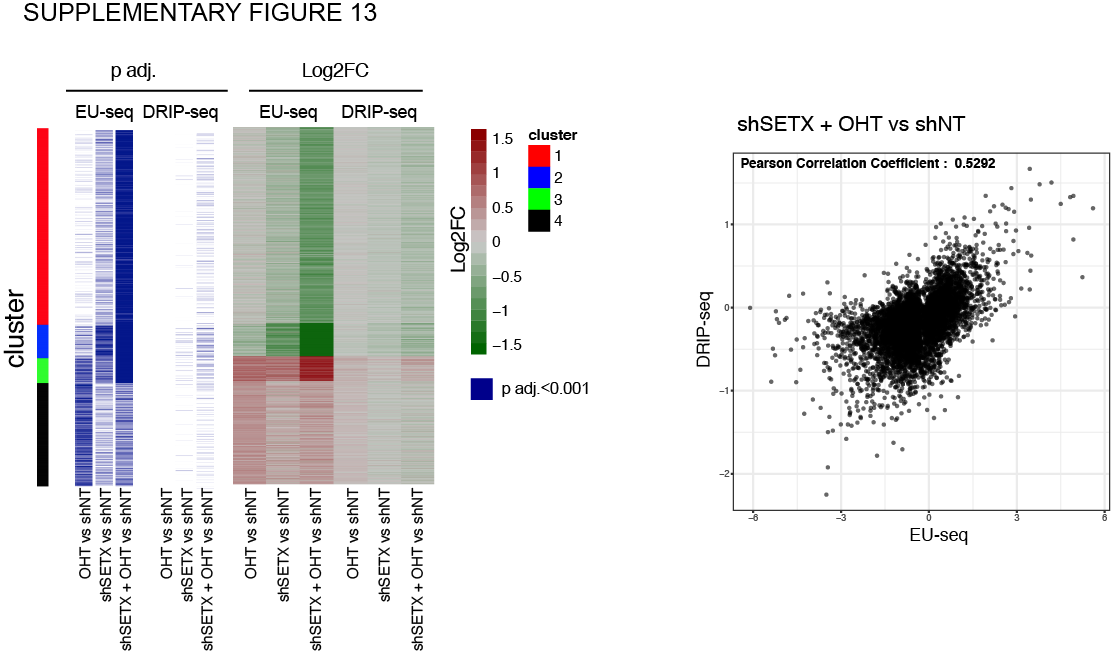
**

**Supplementary figure 13. Genome wide alteration of transcription and R-loops upon SETX silencing and MycER activation.** (a) Unsupervised clustering based on EU-seq and DRIP-seq. (b) correlation of EU-seq and DRIP-seq log2FC in shSETX-MycER cells compared to mock cells.

**
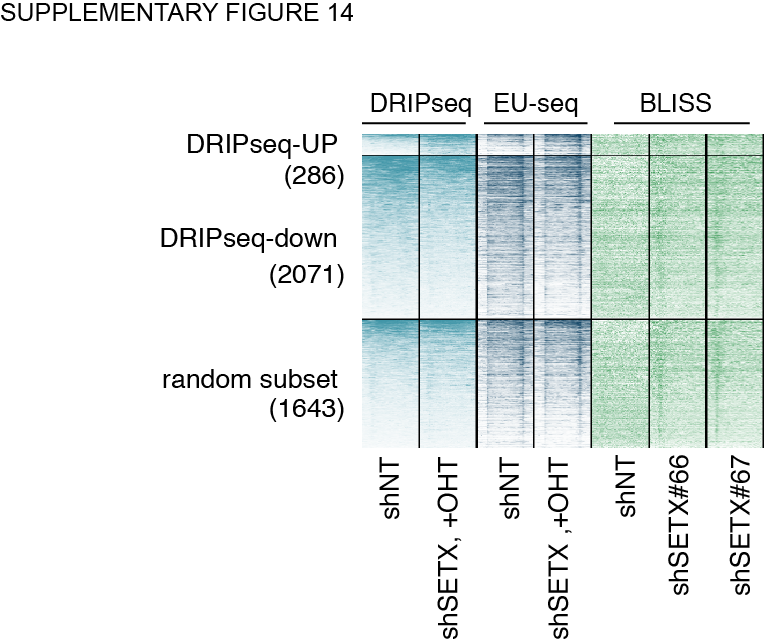
**

**Supplementary figure 14. Analysis of genes showing altered RNA-DNA hybrids upon MycER activation and SETX silencing.** Heatmaps ranked by DRIP-seq signal reporting the distribution of nascent RNA (Eu-seq) and DSBs (BLISS signal) in control cells (shNT) or upon MycER activation and SETX silencing (shSETX, +OHT).


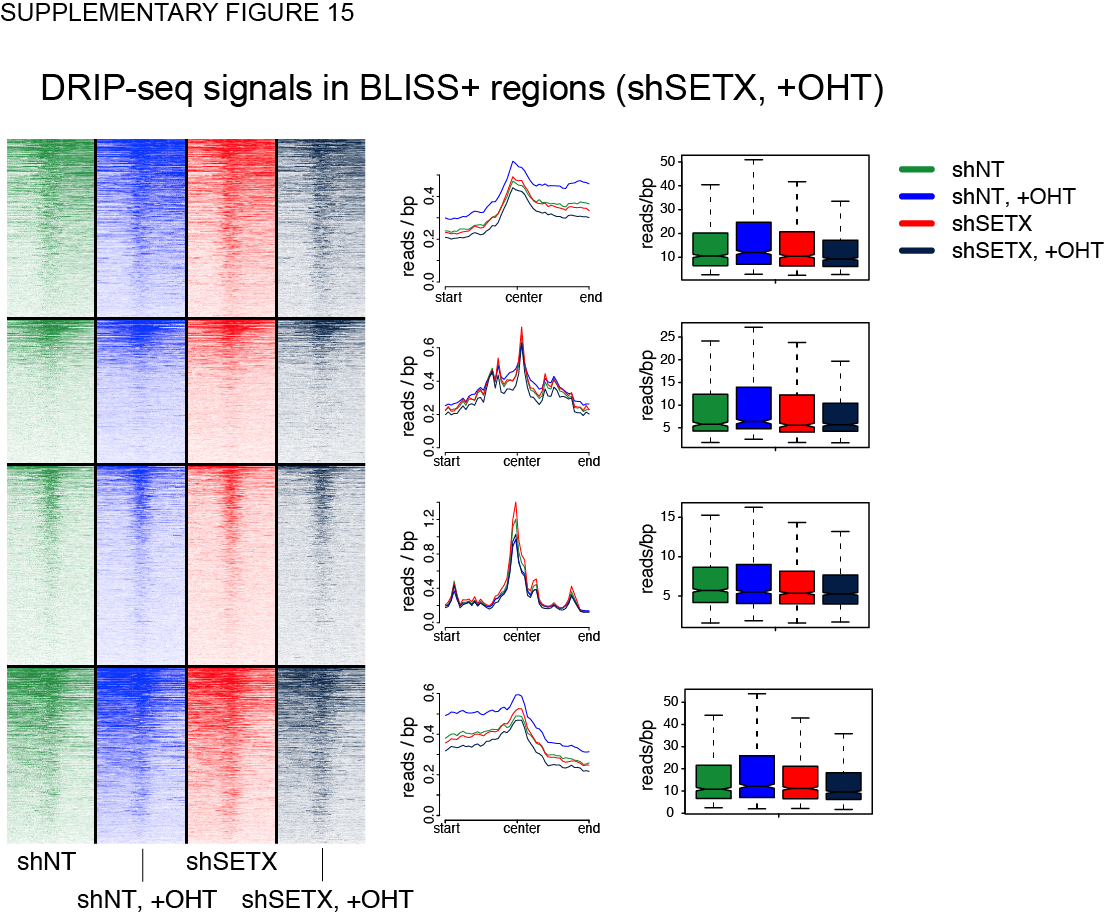


**Supplementary figure 15. Analyses of RNA-DNA hybrids within BLISS+ regions detected upon MycER activation and SETX silencing.** Left, ranked heatmaps of DRIP-seq signals in the BLISS+ clusters shown in figure 7e. Metagene plot (center) and box plot (right) of DRIP-seq signals detected in the four BLISS+ clusters. Heatmaps and metagene plots are centered on the BLISS+ region.

**
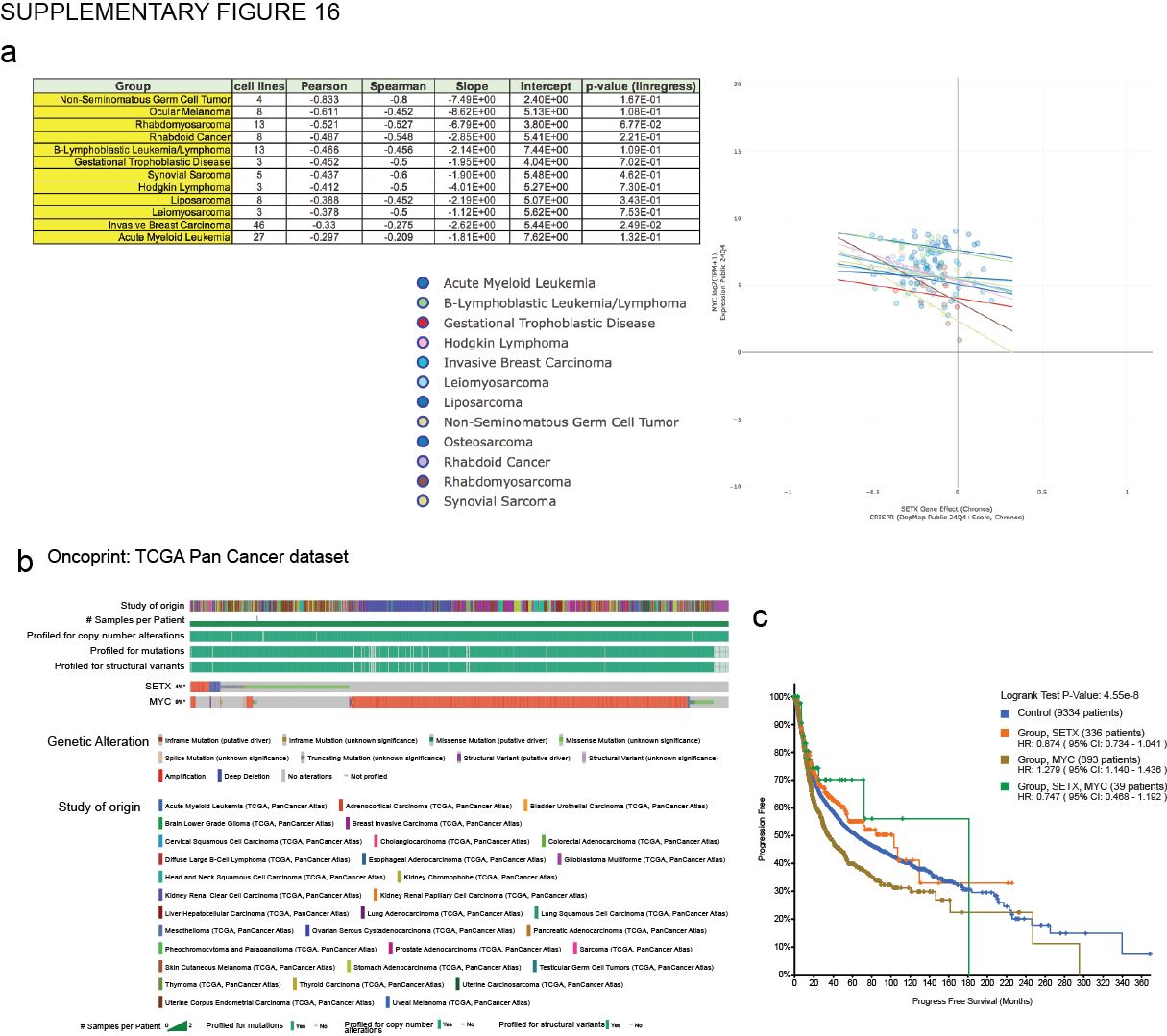
**

**Supplementary figure 16. In silico genomics.** (a) Correlation analysis of MYC expression and the Chronos gene score from DepMap (https://depmap.org/portal/#). The Chronos gene score indicates the lethality of the KO of a given gene, with a negative score indicating higher lethality. Cell lines were subset based on tumor type. The table on the right reports the values and statistics of the regression curves. The plot on the left reports those tumor types for which there is a negative correlation between MYC expression and SETX gene score: these are tumor lines where SETX loss is more lethal in MYC overexpressing cells. (b) Oncoprint reporting mutations of MYC and SETX in the TCGA pan-cancer datasets (cBioPortal). (c) Survival analysis (progression-free) of patients reported in the TCGA pan-cancer dataset. Patients were subset based on MYC or SETX annotated mutations, while the control group comprised patients with no mutation in either MYC or SETX.
