# Supplementary figures and images for: Senataxin prevents replicative stress induced by the Myc oncogene
Source: Cell Death Dis. 2025 Mar 19;16(1):187. doi: 10.1038/s41419-025-07485-4 (PMC11923212; doi:10.1038/s41419-025-07485-4)

Figure 2C

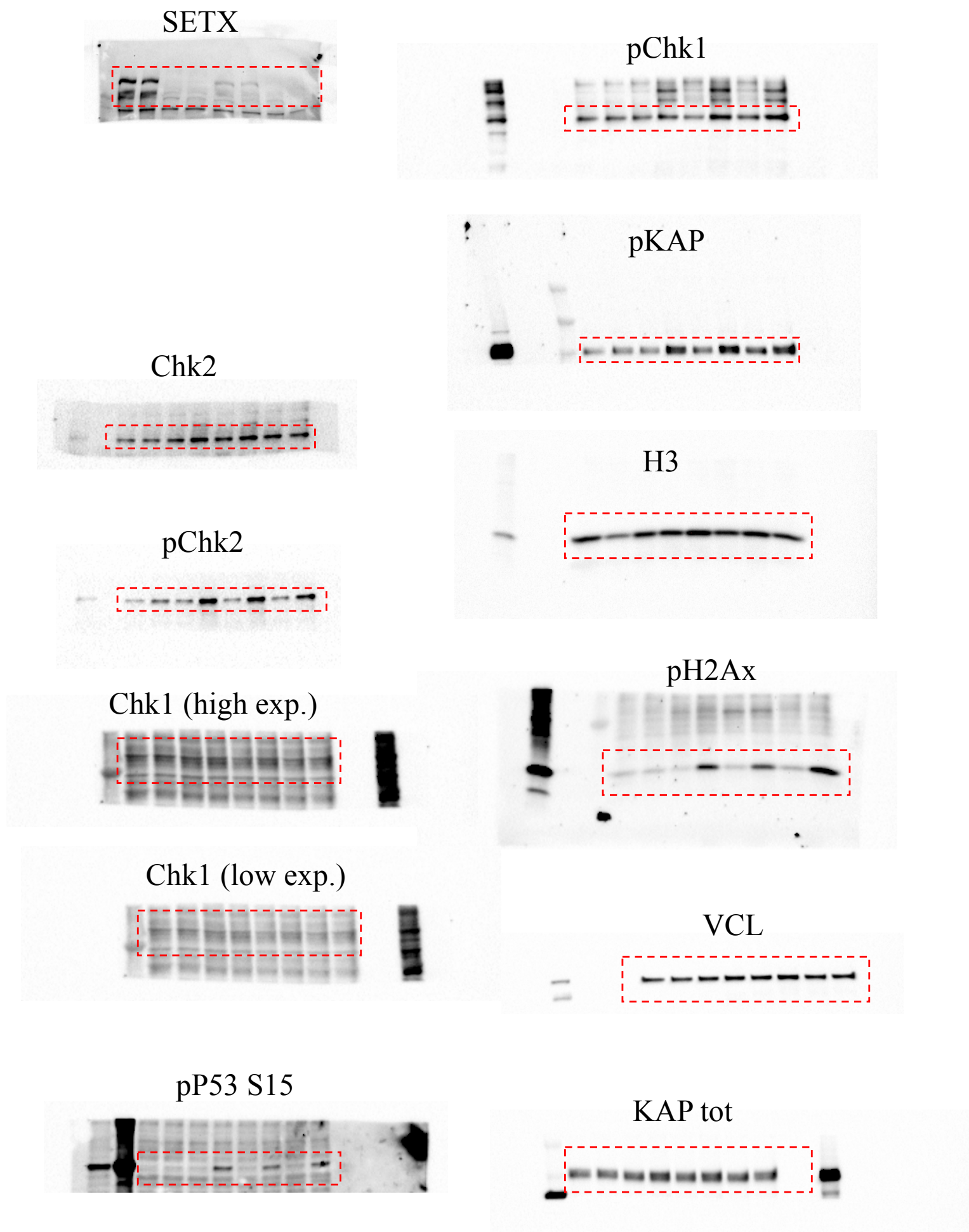

Figure 1e

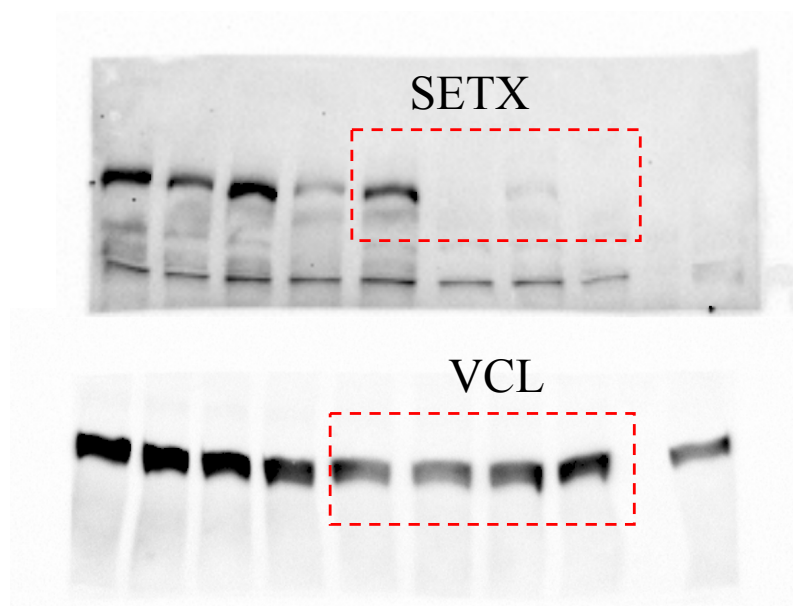

Figure 3e

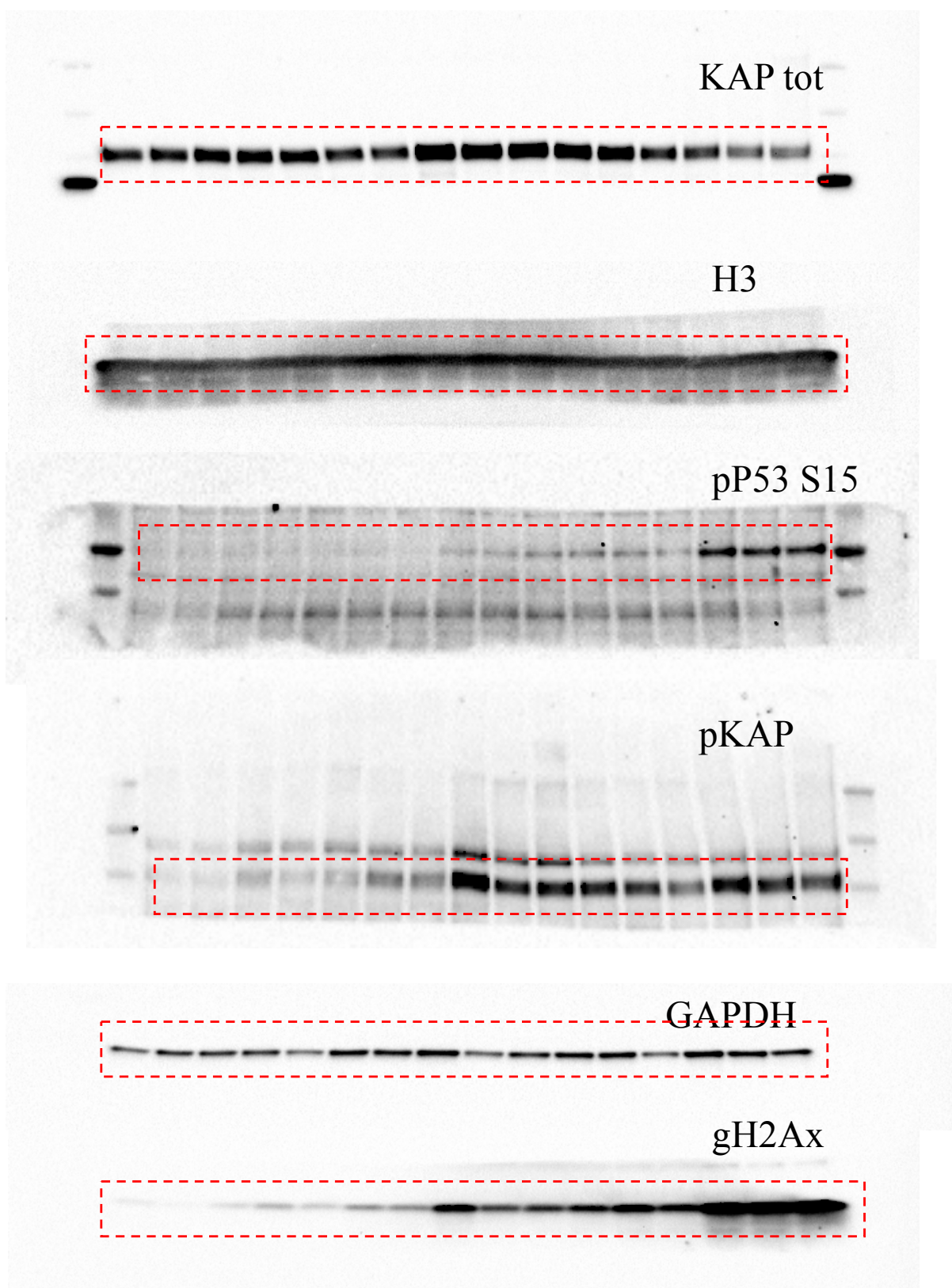

Supplement: Supplementary file 4 — uncropped western blots [file 41419_2025_7485_MOESM4_ESM.pdf]
